# The diversity and consistency of what and when people eat

**DOI:** 10.1038/s42255-026-01504-0

**Published:** 2026-04-23

**Authors:** Tyler Tran, Emily N. C. Manoogian, Zhaoyi Joey Hou, Shweta Varshney, Jialu Sui, Kyla L. Laing, Jason G. Fleischer, Satchidananda Panda

**Affiliations:** 1 The Salk Institute for Biological Studies, La Jolla, CA USA; 2 Department of Cognitive Science, University of California San Diego, La Jolla, CA USA

**Keywords:** Epidemiology, Risk factors, Metabolism

## Abstract

The timing and types of food that people eat, along with associated daily fluctuations, can influence health and wellbeing. However, there are limited data on how eating patterns remain consistent over multiple days. Here we present an exploratory, cross-sectional analysis of over 20,000 adults who recorded more than 2.5 million food logs over 2 weeks using the myCircadianClock app. Our analysis reveals significant variability in food timing and diversity. The time window during which 95% of food and beverages were consumed ranged from 10 h 54 min for the lowest decile to over 16 h for the highest. The median number of unique food and beverage items consumed over the 2 weeks varied from 20 to 86, and only a subset was consistently eaten on multiple days. Many common foods were regularly consumed at specific times of the day, and factors like age, gender and work schedules influenced both eating patterns and food choices. These findings provide a foundation for using longitudinal food records in nutrition and lifestyle research to enhance our understanding of human behaviour and health.

## Abstract (web summary)

An exploratory, observational analysis involving 2.5 million food logs from over 20,000 adults across 2 weeks highlights both consistent and divergent patterns in food habits that may, in turn, influence human health.

## Main

Understanding how the quantity (total energy intake) and quality (macronutrient composition) of dietary intake relate to population health has long been a central focus of public health research and policy. The 7 day food list recall, originally adopted by the United States Department of Agriculture in the early 20th century^1^, laid the foundation for the development of the 24 h dietary recall (24HDR). Among the methods available for capturing dietary behaviours at scale, the 24HDR has emerged as a widely accepted and pragmatic tool^2,3^. Since its formal inclusion in the National Health and Nutrition Examination Survey (NHANES) beginning in 1971–1974, the 24HDR has served as a cornerstone of nutritional surveillance in the United States, enabling population-level assessments of dietary exposures and their associations with health outcomes^4,5^, albeit with certain limitations^6^.

Over the past five decades, considerable methodological refinement has improved the reliability and granularity of the 24HDR. Recognizing that dietary intake varies not only day-to-day but also between weekdays and weekends—often as a result of social and occupational factors—NHANES adopted protocols to collect 24HDRs from both weekday and weekend days^7^. Two or three 24HDRs also better report energy intake than a single 24HDR^8^, and repeat 24HDRs, along with appropriate statistical methods, also improved reporting of nutrient intake by subpopulations^9,10^. To reduce underreporting and enhance recall accuracy, the US Department of Agriculture developed the five-step Automated Multiple-Pass Method, which structures recall by associating food intake with defined eating occasions (for example, breakfast, snack, dinner) and their timing^11,12^. This time-anchoring prompt aids memory and has expanded understanding of the frequency and content of eating events across demographic subgroups. The use of 24HDR in NHANES, the UK Biobank and other population-based cohorts has solidified its role as a core instrument for nutritional epidemiology as well as in prospective studies.

Although originally designed to capture energy and nutrient intake, the 24HDR has also evolved to assess broader dimensions of diet quality, including food group composition and dietary diversity^13–15^. Historically, dietary variety was positively associated with improved nutrient adequacy, reduced mortality and lower risk of chronic diseases, leading to its inclusion in US dietary guidelines for much of the 20th century. Yet in recent decades, this narrative has been challenged^16,17^. Proliferation of foods—over 12,000 new products entering the US market annually—has reframed dietary variety as a potential driver of overeating and metabolic dysregulation^18^. Meanwhile, consumer behaviour research suggests that food choices may be less habitual than what is interpreted from epidemiological data^19^, raising concerns about the validity of single-day dietary assessments in capturing long-term eating patterns.

Emerging evidence from circadian biology now suggests that when food is consumed—its timing relative to the sleep–wake cycle and circadian physiology—has a critical role in metabolic regulation and disease risk^20^. Daily fluctuations in digestion, nutrient absorption, insulin sensitivity and glucose metabolism are all governed by circadian rhythms^21,22^. Consequently, features of an individual’s eating pattern—such as the duration of the daily eating window, its timing relative to sleep and day-to-day variability—may influence cardiometabolic outcomes^23–25^. These insights have prompted a wave of retrospective analyses using 24HDR data to infer temporal eating patterns and their associations with obesity, diabetes and cardiovascular disease.

However, the utility of 24HDR for this purpose remains debated. In NHANES, time-of-day data are used as memory aids rather than validated time stamps, and independent studies have found only modest concordance (*r* = 0.15–0.45) between 24HDR-reported eating times and contemporaneous food diaries^26^. Nevertheless, several analyses assume that a single 24HDR reflects habitual eating windows, often generalizing weekday or weekend patterns across extended periods. As a result, estimates of the average American adult’s eating window vary widely—from 10 h to 13 h—depending on the study^27–29^. Reports have also reached conflicting conclusions on whether shorter eating windows confer cardiometabolic benefit or pose risks, such as increased cardiovascular mortality, particularly when achieved through prolonged morning or evening fasting^30–32^.

The limitations of cross-sectional recall data have become even more apparent considering prospective time-restricted eating (TRE) trials. These studies suggest that compressing the daily eating window to 6–10 h can improve metabolic outcomes^33^. Yet questions remain whether TRE benefits arise from changes in nutrient timing, food choices, caloric intake or circadian alignment. Studies have shown that TRE can inadvertently reduce energy intake and improve nutrition quality, and, given that different foods are preferred at different times of the day, early or late TRE cohorts can also differ in their food choices, potentially confounding associations between meal timing and health outcomes.

Addressing these complex questions requires innovation in dietary assessment methodologies that extend past energy and nutrient intake to capture longitudinal eating behaviours, including timing, frequency and consistency^34^. Web-based tools like ASA24 and UK WebQ have expanded the reach of traditional 24HDR, but they remain constrained by respondent burden and limited time resolution. Smartphone adoption, which now exceeds 90% among US adults, offers new opportunities to capture dietary habits in real-time and real-world settings. However, most of the smartphone apps are developed to promote nutrition quality and quantity^35^.

To meet this challenge, we developed the myCircadianClock (mCC) smartphone application, which enables users to log dietary intake using natural language or images, with automatic timestamping. To minimize food-logging-induced changes in dietary habits, the users are blinded to their data for the first 2 weeks^36^. Following initial validation in smaller cohorts^37^, the mCC app has been deployed in pilot and randomized controlled trials to monitor and modulate eating patterns^38–45^. Our prior study on eating patterns was restricted to mostly healthy adults living in the San Diego area^37^. Shift workers and those with any active diet-based weight management were excluded. We discovered that the eating pattern of adults is more erratic and their daily eating pattern spans a window >12 h. Whether the results apply to a wider cohort and whether the eating pattern and food choices interact were unknown. To explore real-world feasibility and scalability, and to address how age, sex and work type affect eating patterns and food choices, we launched a public version of the app, allowing over 21,000 adults (age of ≥18 years) to log their habitual dietary patterns over a 2 week period.

In this study, we present an analysis of these self-reported data, focusing on the consistency of eating windows, variability in food choices and demographic influences such as age, gender and occupation. Our findings underscore the heterogeneity of eating behaviours and demonstrate the feasibility of leveraging mobile technology to capture the temporal dimensions of diet at scale. These insights offer a foundation for more nuanced and temporally aware nutrition research and interventions.

## Results

### Participants and dietary records

We analysed food and beverage (f/b) logs from adults (age of ≥18 years) who digitally consented to use the myCircadianClock app^36^ to record all their ingestive behaviours for 2 weeks. To minimize the potential effects of diet self-monitoring on dietary habits^46^, the users could not review their logs and were not given any feedback or advice about their diet quality, quantity or timing during this 2 week period. They received a daily reminder to record their dietary intake. Participants were asked to log all their ingestion events in real time with a simple log in natural language and were encouraged to take a picture of the food or beverage. The log was time-stamped by the phone with a ‘log time’ and transmitted to the server, where it was recorded with a timestamp of the server (‘server time’). If the participant forgot to log in real time, she or he could log the food and enter an approximate time of consumption (log time). In case of poor or no internet or Wi-Fi signal, the log waited in the phone until transmission to the server was possible. The time when the log reached the server was also recorded as server time. The time difference between the log time and server time reflected whether the food log was recorded in real time or was delayed as a result of retrospective user reporting or a delay in transmission from smartphone to server. We estimated that 93.54% of logs were recorded in real time on the smartphone device.

**Fig. 1:**
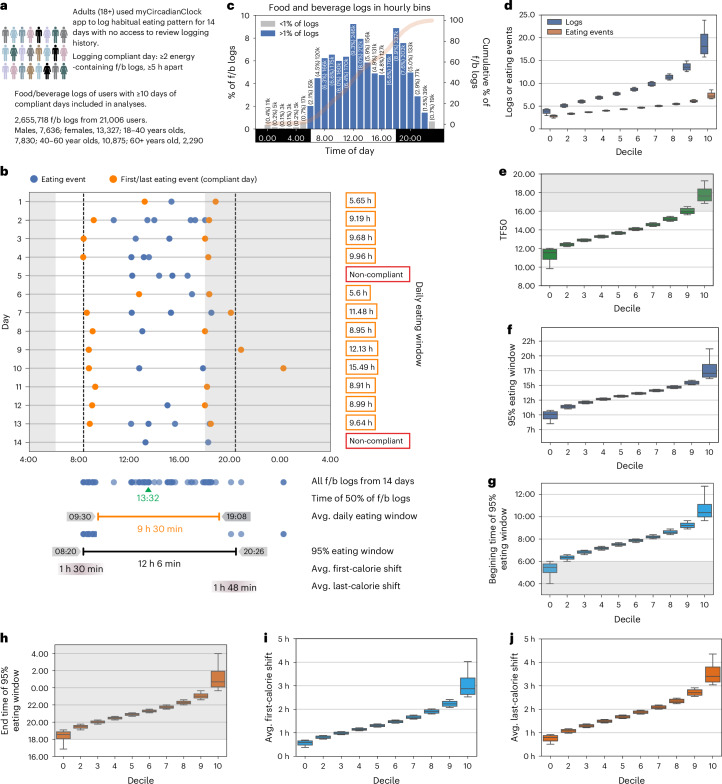
Eating pattern from 14 days of food records. **a**, Schematic of data collection from the myCircadianClock smartphone app. **b**, Representative record of 2 weeks of food and beverage consumption from a user and various parameters describing eating patterns discussed. **c**, Frequency distribution of food and beverage logs recorded in hourly bins. **d**, Number of daily food logs and eating occasions (groupings of food logs). **e**, Median time of all food logs (TF50). **f**, Length of 95% eating window. **g**, Start time of eating window. **h**, End time of eating window (*n* = 21,006). In **b**, **e**, **g** and **h**, night (6 pm–6 am) is shaded grey. **i**, Day-to-day first-eating event shift. **j**, Day-to-day last-eating event shift from each decile (~2,100 users per decile bin) bin of users. For **d**–**j**, the deciles were calculated independently for the respective parameters. Plot whiskers range from 2.5th to 97.5th percentiles, and the boxes display interquartile range (IQR) and median using data from all users (*n* = 21,006).

Reliability of timing of breakfast and dinner increases with repeated 24 h recalls and reaches >75% after at least nine 24HDRs^47^. Prior studies using the same app found that individuals who consent to log all their ingestive behaviours for 2 weeks on average log for at least 10 days with more than two calorie-containing food or beverages per day, ≥5 h apart^38,41^. Using this criteria, there were 21,006 adults (7,621 males, 13,321 females; demographic data in Supplementary Table 1) who recorded 10–14 days of food and beverage records (total number of f/b logs, 2,655,718; mean (±s.d.) number per person, 126.43 ± 62.91; males, 122.38 ± 62.64; females, 128.71 ± 62.94), and these data were used for further analyses (Fig. 1a,b).

Prior sleep studies have shown that many people can go to bed after midnight^48^, and human activity reaches a trough between 02:00 h and 04:00 h^49^; therefore, 04:00 h to 03:59:59 h the next day was considered the beginning and end of the 24 h day. The frequency of f/b logging (individual entries), as seen earlier with smaller cohorts^37,39,40,50^, showed a daily rhythm with low consumption (<1% of all logs per hourly bin) between 23:00 h and 05:00 h and two peaks at 12:00 h and 18:00 h local time (9.29% and 8.93% of all records, respectively) (Fig. 1c). A total of 20.35%, 40.58%, 35.95% and 3.12% f/b records were logged in the first (04:00–10:00 h), second (10:00–16:00 h), third (16:00–22:00 h) and fourth (22:00 04:00 h) quartile of the 24 h day (Fig. 1c and Supplementary Table 2). F/b entries logged within 15 min of each other were considered an eating event^37,50^. The bottom decile (~2,100 people per decile bin) logged 4.57 records within 3.08 eating events per day, while the top decile logged over 15.29 records within 6.50 eating events (Fig. 1d and Supplementary Table 3). These population-level results are comparable to those observed in 1–5 days of food records from other studies, such as NHANES data^51^.

### Daily eating pattern

**Extended Data Fig. 1:**
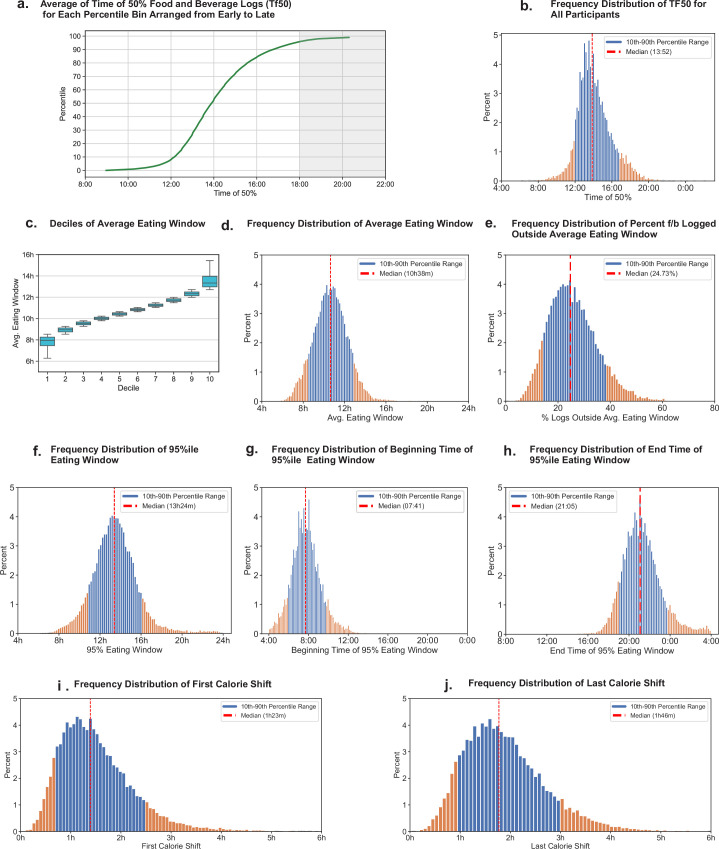
Participant Eating Patterns. **a**. Percentile rank of time of 50% log for all users arranged from earliest (left) to latest (right). **b**. Frequency distribution of TF50 for all users. **c**. Frequency distribution and **d**. mean (+/- sd) of decile bins of users’ average eating window over 14 days. Plot whiskers range from 2.5th to 97.5th percentiles. **e**. Frequency distribution of the percentage of the timing of food/beverage logs that fall outside of the average eating window calculated from the average eating window of compliant days. This shows that the average eating window can miss as much as 26% of the timing of food and beverage consumption. Frequency distribution of **f**. 95% eating window, **g**. beginning of 95% eating window, **h**. end of 95% eating window, **i**. first eating event shift and **j**. last eating event shift.

A raster plot of all energy-containing food logs over 14 days from a randomly selected participant (Fig. 1b) shows that the eating pattern is not consistent and can be described by various metrics. We used multiple metrics to assess daily eating patterns, each providing a unique aspect of the eating patterns of each participant (Supplementary Tables 4 and 5). The time by which 50% of f/b consumption has occurred (TF50) is one of the measures for distinguishing early versus late eaters; early eaters reach this median time earlier than late eaters^52,53^. The TF50 of all f/b records for each user ranged from 12:07 h for the earliest decile to 16:40 h for the latest decile, with a median at 13:52 h (Fig. 1e and Extended Data Fig. 1a,b). However, the TF50 does not reflect the width of the window of time when people eat. The average daily eating window (the mean time between the first and last caloric intake of each compliant day; Fig. 1b) ranged from 8 h 32 min for the first decile to 12 h 43 min for the last decile, with a mean of 10 h 40 min (s.d., 101 min) (Extended Data Fig. 1b,c). However, because of day-to-day variation in eating time, including variation in the first and last eating event, ~25% of f/b logs were recorded outside the average eating window (Extended Data Fig. 1d), demonstrating the limitation of this metric. Another measure of the eating window that captures this day-to-day variation of eating times is the 95% eating window^37^, which is the time between the 2.5th percentile of all f/b logs (that is, excluding a few exceptionally early f/b logs) and 97.5th percentile of f/b log time (that is, excluding a few exceptionally late f/b logs) over multiple days. The mean 95% eating window was 13 h 29 min (s.d., 134 min), ranging from 10 h 54 min (bottom decile) to 16 h 0 min (top decile) (Fig. 1f and Extended Data Fig. 1f). The mean start of the 95% eating window was 07:47 h, ranging from 06:00 h (bottom decile) to 09:39 h (top decile) (Fig. 1g and Extended Data Fig. 1g). The mean end of the 95% eating window was 21:16 h, ranging from 19:05 h (bottom decile) to 23:39 h (top decile) (Fig. 1h and Extended Data Fig. 1h). Given that the 95% eating window, by definition, excludes only 5% of f/b logs, while the average eating window, on average, excludes 25% of f/b records, for the rest of the paper, we will use 95% window as the ‘eating window’.

**Extended Data Fig. 2:**
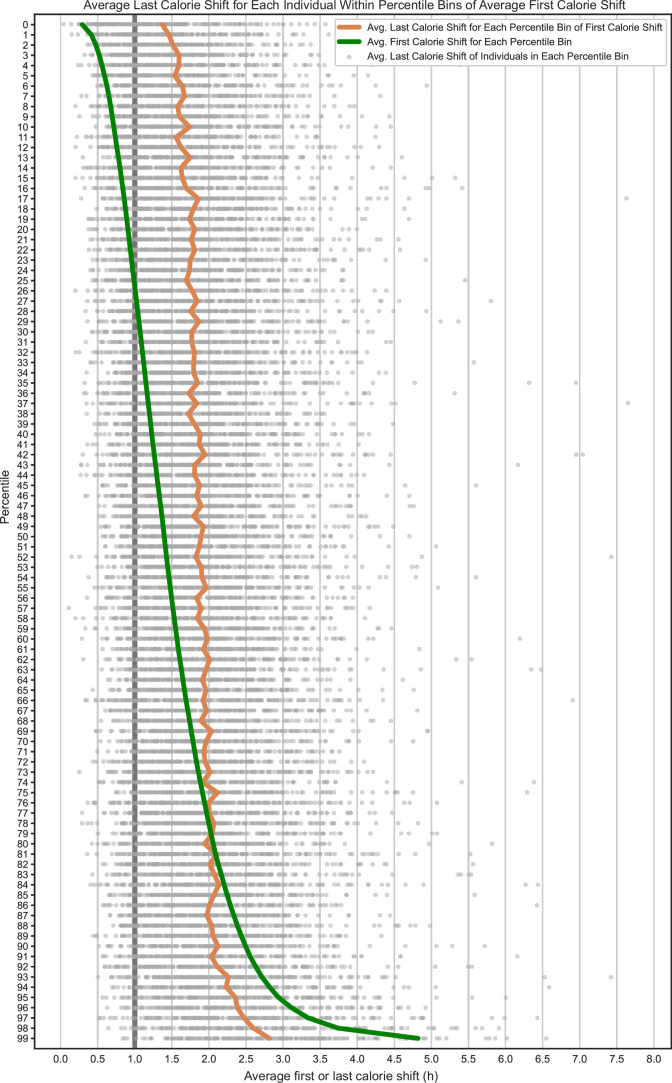
Relationship between first calorie shift and last calorie shift. mean percentile bins of mean first calorie shift (green line) from shortest (top) to longest (bottom). For each percentile bin of first calorie shift, the mean last calorie shift of all individuals in the bin (blue dots) and the bin mean (orange line) are shown. For bins 1- ~75 of first calorie shift, the bin mean of last calorie shift is longer (orange line is to the right of the green line).

**Extended Data Fig. 3:**
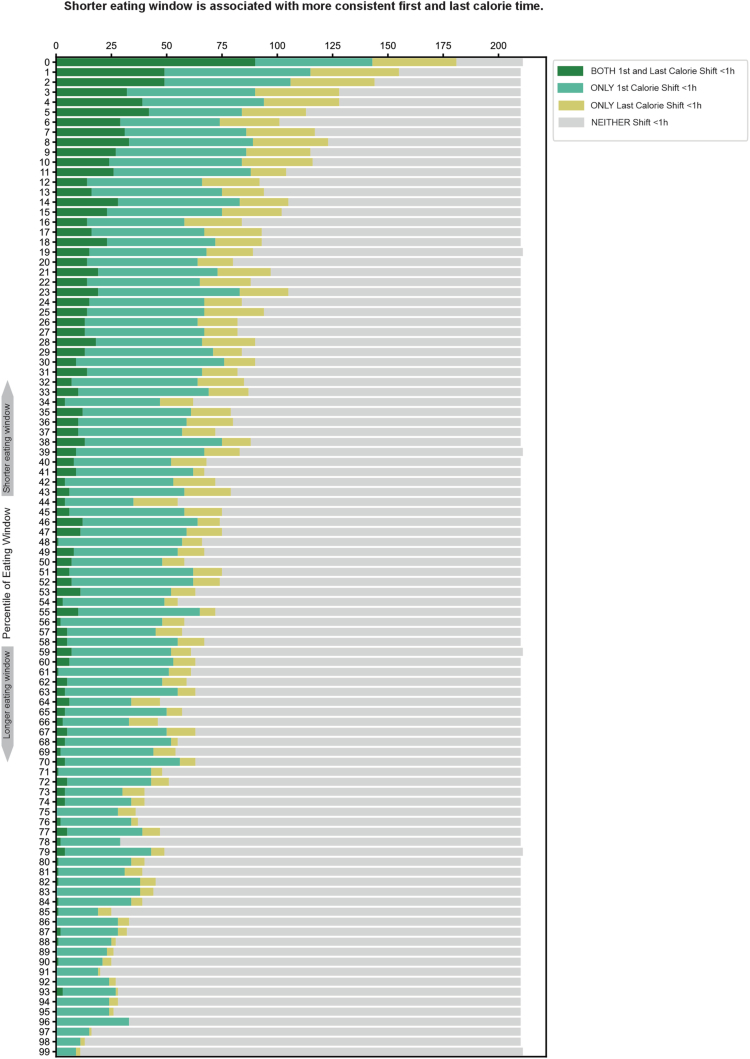
Relationship between eating window and first- or last-calorie shift. For each percentile bin of eating window (~210 individuals/bin; shortest bin on the top to longest at the bottom), the number of individuals who have only the mean first calorie shift <1 h (light blue) or both first and last calorie shift <1 h (dark blue) are shown. Note, bins of shorter eating windows are more likely to have more individuals with smaller first or last calorie shifts and hence are more consistent in their eating window.

Day-to-day variation in the timing of the first eating event contributed to weight change in the CALERIE-II study^54^. For each participant, we calculated the difference between the time of an eating event (for example, the first eating event) on successive days during which the participant was compliant with the logging criteria. A participant’s day-to-day shift is the absolute value of this difference across the 14 day period. The first caloric intake shifted a mean of 1 h 31 min (s.d., 46 min) between successive days across all participants (bottom decile, 0 h 42 min; top decile, 2 h 28 min) (Fig. 1i and Extended Data Fig. 1i), and their last caloric intake shifted a mean of 1 h 53 min (s.d., 50 min) (bottom decile, 0 h 57 min; top decile, 2 h 59 min) between days (Fig. 1j and Extended Data Fig. 1j). Participants were more regular in the timing of their first calorie intake (26.1% had <1 h mean first-calorie shift between successive days) than their last calorie intake (11.9% had <1 h mean last-calorie shift between successive days). Only 5.2% of users were consistent enough in the timing of their daily eating patterns that both first-calorie and last-calorie mean shifts were <1 h (Extended Data Fig. 2). To test any relation between eating window and consistent timing of first or last calorie intake, we calculated the average first-calorie and last-calorie shift for each percentile of eating window. We found that those with a shorter eating window were more likely to have a consistent eating pattern with a first-calorie and last-calorie shift of <1 h (Extended Data Fig. 3).

**Fig. 2:**
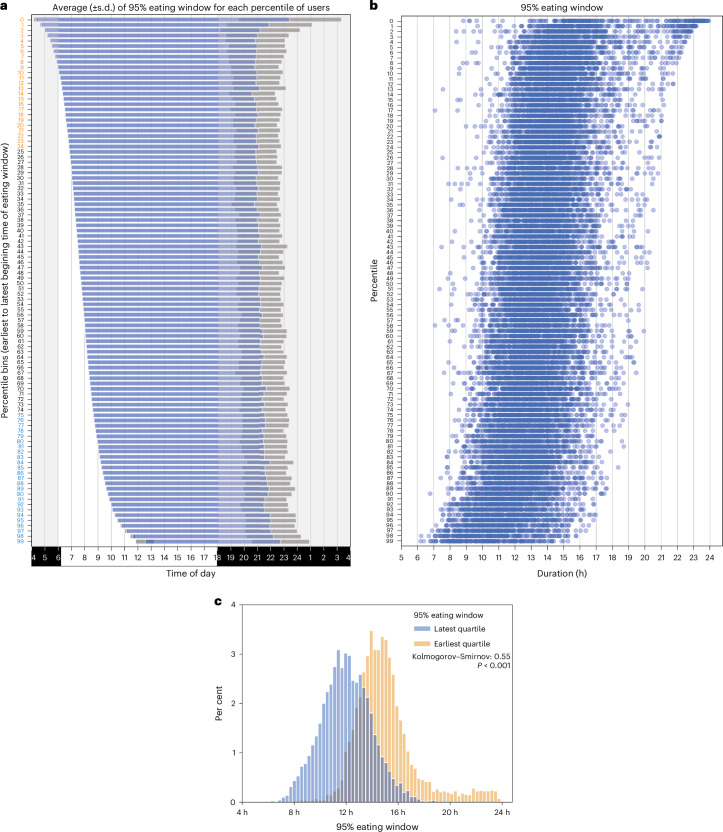
Relationship between the start time of the eating window and the length of the eating window. **a**, Mean of the eating window for each percentile of users (~210 individuals per percentile bin). Grey shading, s.d. Bins are sorted by eating window start time. In **a**, night (6 pm–6 am) is shaded grey. **b**, The eating windows of all individuals in each percentile bin are shown. Each data point represents the eating window of an individual; lower percentiles indicate earlier eating window start times. **c**, The distribution of eating window lengths across quartiles of start times indicates that individuals who begin eating earlier in the day tend to have a longer eating window.

Next, we tested whether when a person starts eating has any effect on the length of the eating window. We ordered the eating window of all participants based on the start time of their eating window, from earliest to latest, and binned them in each percentile (100 bins; each containing ~210 participants) (Fig. 2a,b). The earliest percentile started their eating window at an average of 04:20 h (s.d., 10 min) and had an eating window of 19 h 4 min (s.d., 3 h 55 min). The latest percentile started their eating window at 12:32 h (s.d., 42 min) and had an average eating window of 10 h 11 min (s.d., 2 h 1 min). Similarly, those who begin early (first decile; eating window start time between 04:00 h and 06:00 h) were also likely to have a longer eating window (mean, 15 h 54 min, s.d., 172 min), while those in the top decile or those who start eating later (eating window start time between 09:39 h and 18:07 h) were likely to report a shorter eating window (mean, 11 h 16 min, s.d., 118 min; Mann–Whitney *U* = 4114091.50, *P* < 0.001) (Fig. 2).

### Eating patterns are shaped by work schedule, age and gender

Next, we tested whether age, sex or work type affected eating patterns. The absence of any effect would result in an equivalent number of participants in each decile group for the duration of the eating window, start or end of the eating window, TF50 and first-meal or last-meal shifts. Older participants were more likely to have a shorter eating window and finish their eating window earlier than younger participants (Supplementary Table 6). We found that shift workers were more likely to have irregular eating patterns, marked by longer eating windows and larger first-meal or last-meal shifts.

**Fig. 3:**
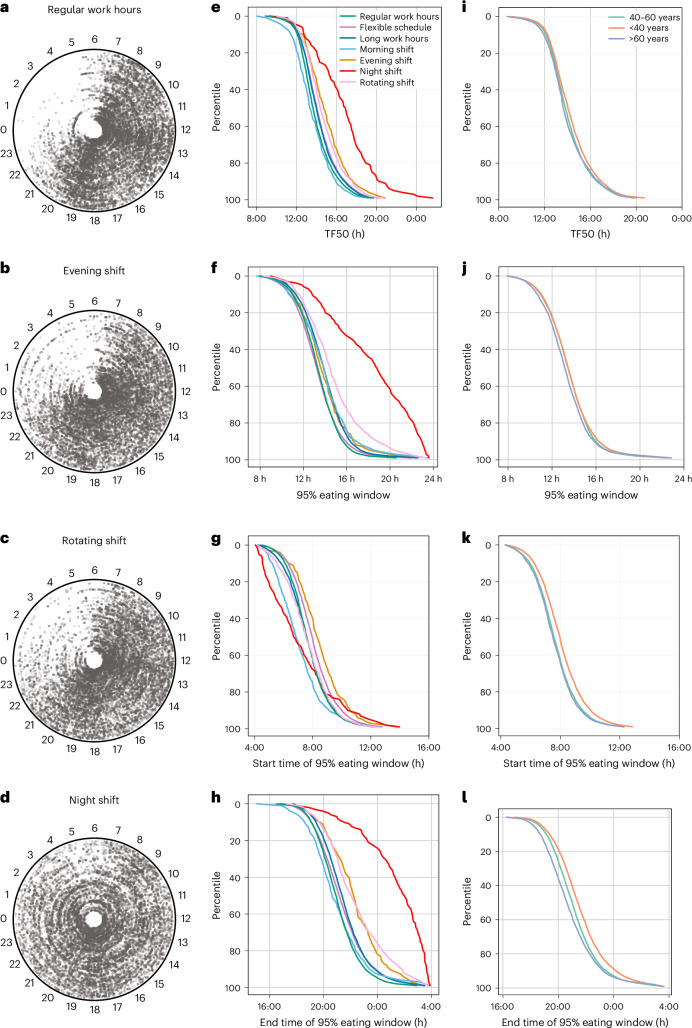
Effects of work schedule, sex and age on eating patterns. **a**–**d**, Polar (clock-style) plots of food and beverage logs for 150 randomly selected individuals who self-identified their work schedule as regular hours (**a**), evening shift (**b**), rotating shift (**c**) or night shift (**d**). Angle around the circle corresponds to clock time, and each concentric circle represents one user. Each point represents the timing of a food log; denser regions indicate peak eating times. **e**, Mean TF50 values arranged from earliest to latest of users from each work schedule shows night-shift workers have remarkably late TF50. **f**–**h**, Percentile rank of eating window (**f**), beginning of eating window (**g**) and end of eating window (**h**) of different work schedules. **i**–**l**, Percentile rank of TF50 (**i**), average eating window (**j**), beginning of eating window (**k**) and end of eating window (**l**) of different age groups.

**Extended Data Fig. 4:**
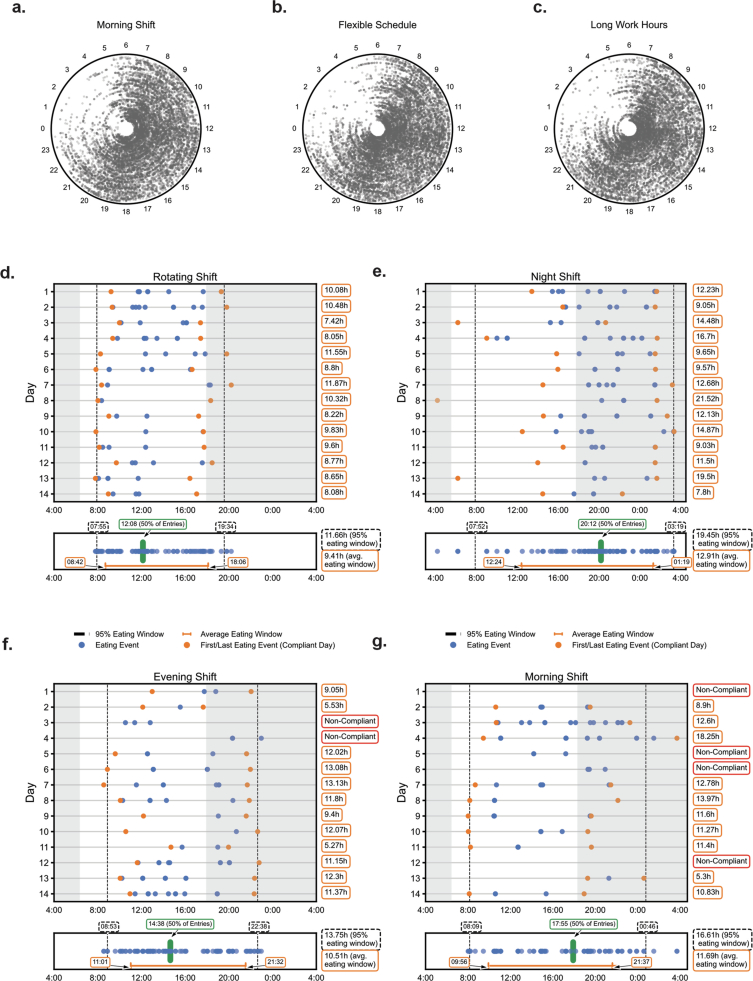
Eating pattern patterns of regular workers and shift workers. Polar plots of randomly picked 150 individuals self-reported as working in **a**. morning shift, **b**. flexible schedule, **c**. long work hours. Representative feedograms of **d**. rotating shift, **e**. night shift, **f**. evening shift, and **g**. morning shift worker.

We further explored whether different types of shift work, self-described flexible schedules or long work hours affected eating patterns. In a visual display of all eating occasions of 150 randomly selected participants with different work types, the eating occasions of night-shift workers were more dispersed throughout a 24 h period than those of individuals who worked regular hours (Fig. 3a–d and Extended Data Fig. 4). Morning-shift workers reached their median f/b log time earliest (mean, 13:30 h, s.d., 116 min) followed by those who worked regular hours (mean, 13:54 h, s.d., 107 min), long work hours (mean, 14:11 h, s.d., 109 min), flexible schedules (mean, 14:14 h, s.d., 114 min), rotating shifts (mean, 14:38 h, s.d., 118 min), evening shifts (mean, 14:51 h, s.d., 130 min) and night shifts (mean, 16:39 h, s.d., 182 min) (Fig. 3e, Extended Data Fig. 4 and Supplementary Table 7). The eating window and the end of the eating window also differed significantly between some of the groups (Fig. 3f–h and Supplementary Table 7). The 95% eating window of self-described flexible workers was close to that of the individuals who worked regular hours. Night-shift workers had the longest 95% eating window (mean, 18 h 4 min, s.d., 234 min), largest mean day-to-day shift in first eating events (2 h 58 min, s.d., 98 min) and last-eating events (mean, 2 h 49 min, s.d., 72 min), and they were significantly different (Supplementary Tables 7–10) from that of regular schedule workers (95% eating window mean, 13 h 19 min, s.d., 118 min; first-eating event shift mean, 1 h 26 min, s.d., 41 min; mean last-eating event shift mean, 1 h 50 min, s.d., 47 min), who exhibited shorter and more stable eating windows than all shift worker groups.

The daily pattern of sleep and wakefulness is also known to change across age^55^, albeit modestly, compared to shift work, which in turn influences eating event timing^56^. Accordingly, we found that younger individuals started their first eating event later (<40 years old; *n* = 7,830; mean beginning of the 95% window, 08:01 h, s.d., 91 min) versus older individuals (>60 years old; *n* = 2,290; mean beginning of the 95% window, 07:34 h, s.d., 85 min). Younger individuals also reached their TF50 later (mean, 14:16 h, s.d., 116 min vs 13:57 h, s.d., 115 min) and had a longer eating window (mean, 13 h 33 min, s.d., 137 min vs 13 h 16 min, s.d., 137 min) than older individuals (Fig. 3i–l and Supplementary Table 11). The middle-aged group (40–60 years, *n* = 10,875) was intermediate in many of these measures (mean eating window, 13 h 30 min, s.d., 131 min; mean start of eating window, 07:39 h, s.d., 86 min; mean TF50, 14:02 h, s.d., 110 min). However, the first-eating event shift did not change remarkably between the young, middle-aged or older age groups (mean, 1 h 32 min, s.d., 48 min, 1 h 30 min, s.d., 44 min and 1 h 31 min, s.d., 44 min, respectively). The last-eating event shift reduced modestly from young to older age groups (mean young, 1 h 55 min, s.d., 49 min; middle-aged, 1 h 53 min, s.d., 50 min; old, 1 h 49 min, s.d., 50 min). Within each of these age brackets, the males and females also showed some differences (Supplementary Table 11).

### What people eat

**Fig. 4:**
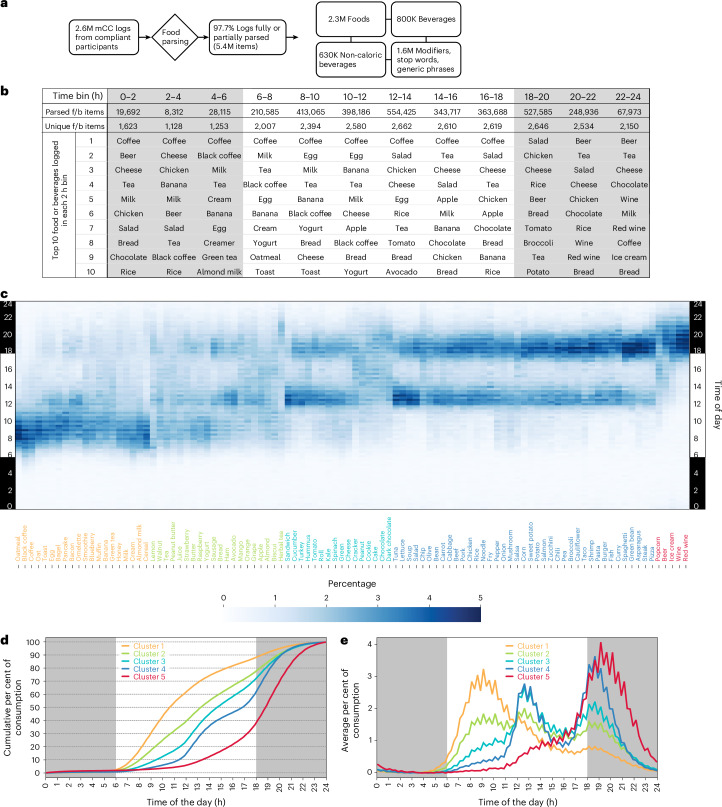
Food preference as a function of time of day. **a**, Schematics of parsing food and beverage logs to individual food and beverage items. **b**, Diversity and popularity of food and beverages in each 2 h bin. The top ten most popular items in each bin are shown in rank order. The total number of items in each bin and the number of unique items in each bin are shown. **c**, Heatmap of hierarchically clustered top-100 food and beverage items and their timing of consumption in 15 min bins over 24 h. The time of day (*y* axis) and the names of the items (*x* axis) are colour-coded for the respective cluster. **d**, Cumulative consumption of items in five clusters is shown. **e**, The timing of consumption of items in five different clusters in 15 min bins shows distinct consumption patterns: cluster 1 is mostly preferred at breakfast, cluster 2 is throughout the day, clusters 3 and 4 are preferred at lunch and dinner and cluster 5 is largely after dinner.

Participants used natural language in their f/b logs to describe food and beverages consumed. Therefore, we used food and beverage names to analyse what people eat, as they are more relatable in real life. The f/b logs recorded in natural language were heterogeneous in content and description; one log may describe only one food item (for example, warm muffin), while some may contain multiple items (latte, croissant and leftover pizza). Each f/b log was parsed by a custom parsing pipeline to extract food or beverage names and excluded generic terms (for example, breakfast, lunch, dinner, Chinese food and so on) or stopwords (for example, the, a) and some modifiers (for example, leftover, warm, grilled, spicy). As has been done in other studies, such as re-analyses of NHANES records^57^, similar foods or beverages were also assigned one name (for example, bread, whole wheat bread and whole grain bread were grouped as ‘bread’). This yielded 2,227 foods and 530 beverages in the library. The logs from 21,006 users yielded 2.3 million foods and 800,000 beverages that matched a common name in the library (Fig. 4a). A total of 97.7% of logs contained items that could be matched to common names (Fig. 4a). We assessed how more than three million parsed items representing 2,757 foods and beverages were consumed across a 24 h period.

As expected, for the entire cohort, the diverse types of food logged at different times of the day varied. Only 8,312 parsed items representing 1,128 unique f/b were logged between 02:00 h and 04:00 h, while 554,425 items (2,662 unique f/b) were logged between 12:00 h and 14:00 h, and 527,585 items (2,646 unique f/b) were logged between 18:00 h and 20:00 h (Fig. 4b). For each 2 h bin, we calculated how many times each unique f/b item was consumed. Food consumption frequency (total logging frequency in 2 h bins) revealed the top ten items across all 12 bins. Many items appeared repeatedly in the top ten items for each 2 h bin, resulting in 28 unique f/b making up the top ten list in 12 bins. Tea was among the top ten most frequently consumed items throughout 24 h. Other items remained among the top ten for one specific window or a prolonged window of time (for example, coffee from 0:00 h to 16:00 h, eggs from 04:00 h to 12:00 h, beer from 18:00 h to 04:00 h), while others, such as oatmeal, were popular only during one or more bins (Fig. 4b).

Out of the 2,757 unique f/b, only 788 items (670 foods, 118 beverages) were consumed at least once by >1% of the cohort (~210 participants). Among the rank order of frequently logged items, the top-100 items included 86 foods and 14 beverages (Fig. 4c and Supplementary Table 12). The most popular item—coffee, a beverage—was consumed by 71.91% of users, while the most popular food—salad—was consumed by 66.69% at least once in 14 days. The 100th most popular item—muffin—was consumed by 12.28% of users at least once in 14 days. Other notable popular items included egg as the third most popular item (logged by 64.01% of users), chicken as the fourth (63.38%), rice at sixth (52.58%), bread at ninth (45.96%), tea at 13th (41.60%), pasta at 20th (34.09%), beef at 39th (25.38%) and pork at 54th (18.53%).

**Extended Data Fig. 5:**
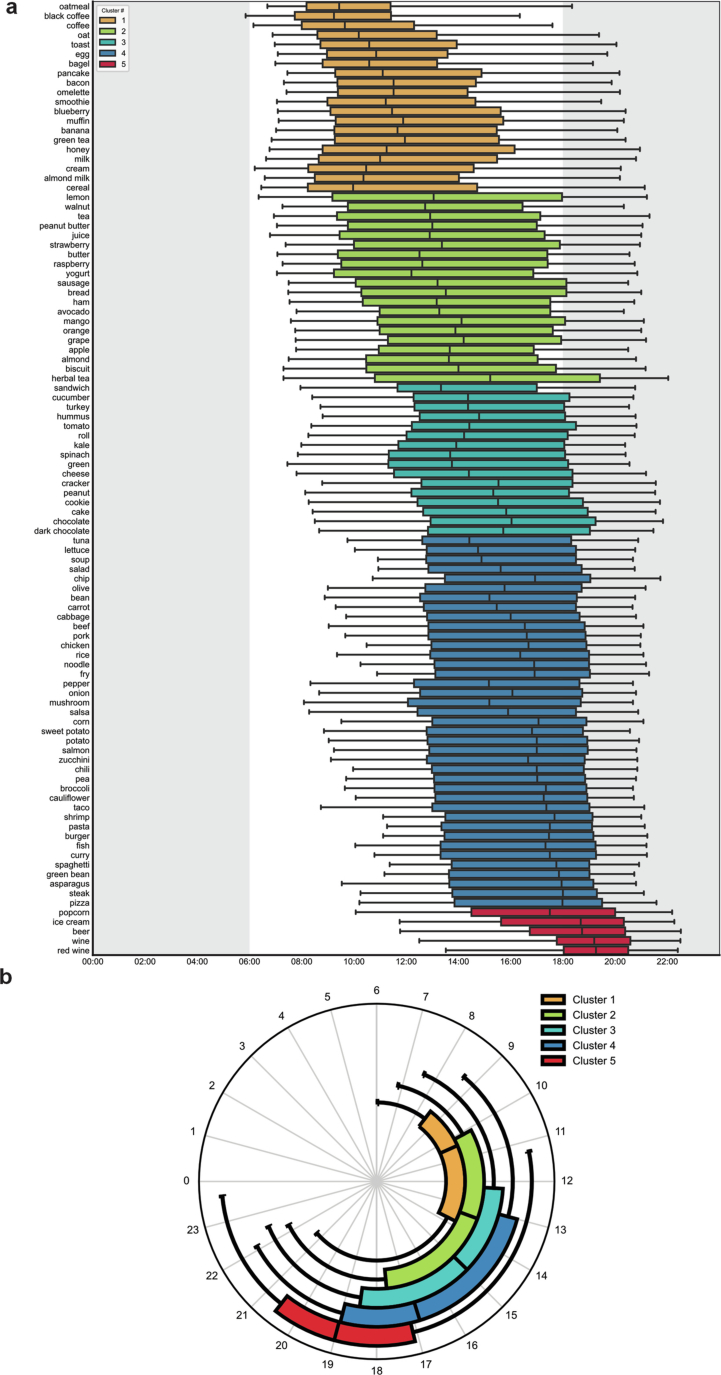
Time of consumption of top 100 items. **a**. Box plots representing median (25-75% range) and whiskers representing 10-90% range for each of the 100 items item groups by cluster clusters 1 through 5. **b**. Box plots along the radial axis showing the median (25-75% range) and whisker representing 10-90% range for each of the five clusters in the top 100 food and beverages.

At least one of the top-100 popular items was consumed at least once by 99.94% of all users and represented 57.01% of all f/b items logged. Owing to the over-representation of these items in food records and their consumption by nearly all participants, we focused on their daily consumption patterns. Hierarchical clustering of these top-100 items and their consumption time produced five dominant clusters (Fig. 4c and Extended Data Fig. 5). For each of these top-100 items, we also calculated their TF50. Cluster 1 (20 items: 12 foods and eight beverages) appeared to be the morning cluster, containing several typical breakfast items (for example, cereal, pancake, oatmeal, bagel, muffin, milk, bacon, blueberry) whose consumption reached TF50 on average by 10:51 h (Fig. 4d,e and Extended Data Fig. 5). Cluster 2 items (20 items: 17 foods and three beverages; for example, bread, butter, peanut butter, yogurt, grape, orange, mango, juice, tea) were consumed almost throughout the day, with major peaks around breakfast and lunch and a minor peak around dinner. These items reached their TF50 at 13:28 h. Cluster 3 (16 items: 16 foods and zero beverages; for example, sandwich, hummus, chocolate, spinach) and cluster 4 (39 items: 39 foods and zero beverages; for example pasta, spaghetti, noodle, burger, rice, curry, taco, fish, steak, pizza) were mostly consumed at lunch and dinner. Cluster 3 was slightly more popular around lunch and reached TF50 at 14:50 h, while cluster 4 was dominant over cluster 3 at dinner time and reached TF50 around 16:46 h. Cluster 5 was the smallest, with five items (popcorn, beer, ice cream, red wine and wine), whose consumption started rising at lunch, attaining TF50 at 18:48 h and reaching peak after all other clusters peaked.

**Fig. 5:**
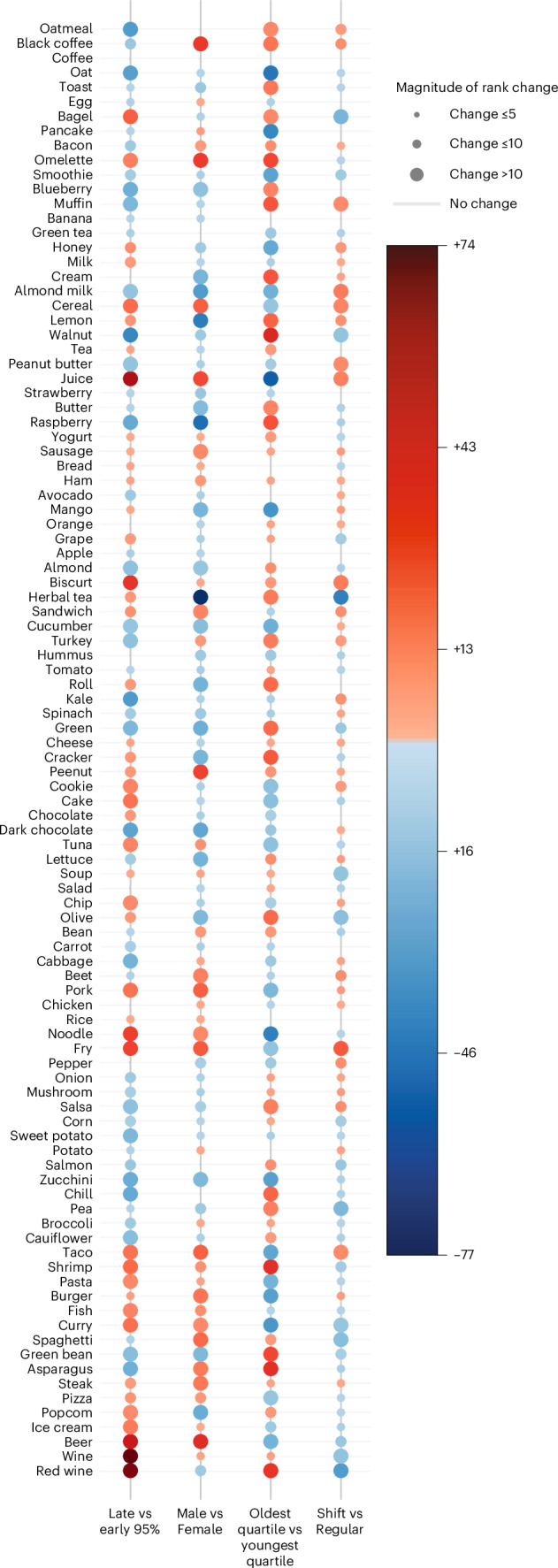
Change in rank of top-100 items between groups (bottom labels). For each pair of compared groups, items climbing up in rank or preferred by group 1 are in blue circles, and those sliding in rank or less preferred are represented in red circles. The size of the circle and intensity of colour represent the magnitude of change.

This rank order of 100 food items was determined for the entire cohort, so next, we asked whether food popularity is affected by sex, age, work schedule and preferred timing of eating. To quantify overall differences in the preference of these top items between subgroups, we computed the Jensen–Shannon distance (JSD), which measures the dissimilarity between two probability distributions. Specifically, we calculated the JSD between subgroup-level item popularity distributions, defined as the percentage of users in each subgroup that logged each item. Although the observed JSDs were modest (male vs female, 0.07; early vs late eaters, 0.07; youngest vs oldest, 0.07; shift vs standard work schedule, 0.03), all exceeded chance expectations in permutation testing (1,000 permutations, *P* = 0.001). As a global summary measure, JSD reflects aggregate dissimilarity but does not reveal the specific foods or beverages that differ in prevalence between groups. Therefore, we interrogated the direction and magnitude of change of rank order (upward increase in rank order = more preferred) (Fig. 5 and Supplementary Table 13). Although some of the top-100 items were less affected by these factors, showing minimal rank order change (for example, coffee, bread, rice, chicken), a subset including cereal, juice, tacos and fries shifted substantially (>10) between groups. Young versus old participants showed the greatest number of such differences (47 items), followed by early versus late eaters (44 items), males versus females (37 items) and shift versus regular workers (19 items). Group-specific preferences included almond milk, dark chocolate and herbal tea among females, and black coffee, beer and steak among males. This observed gender difference is comparable to what was reported earlier^58^. The youngest quartile favoured pancakes, burgers and juices, while older adults preferred red wine, walnuts and crackers. Shift workers (all types, *n* = 1,541) preferred fries, cereal and almond milk, although with typically lower rank shifts than other group comparisons. We also found that early eaters—those who ended their 95% eating window before 19:00 h (*n* = 1,860 users)—prefer walnuts, dark chocolate and oatmeal, while those 95% eating window ended after 22:00 h (*n* = 6,278 users) prefer beer, wine and cereal. Interestingly, all cluster 5 foods and beverages (popcorn, ice cream, beer, wine and red wine) ranked higher among late eaters.

**Fig. 6:**
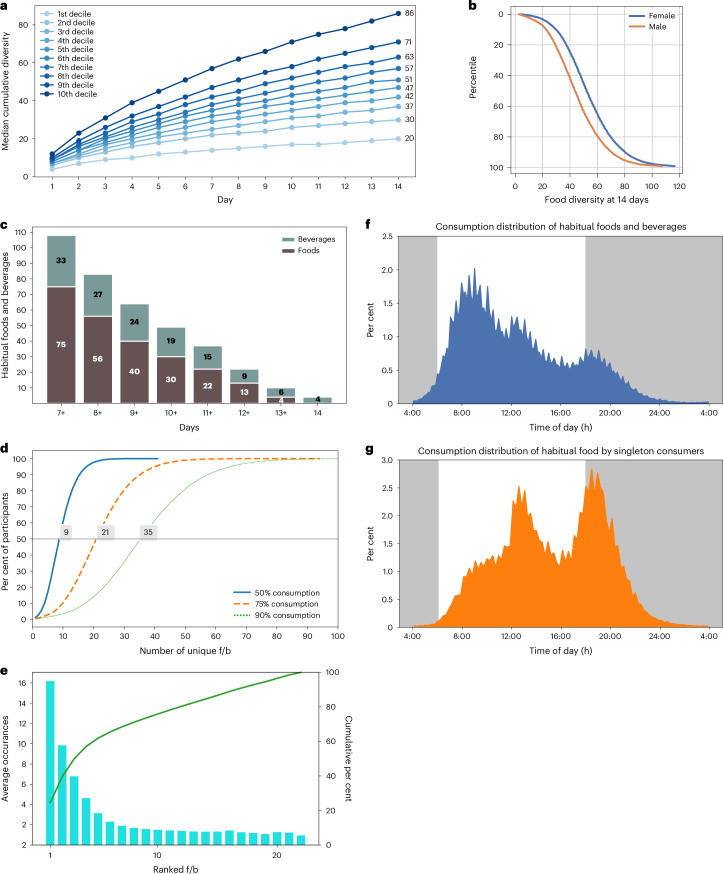
Diversity of foods consumed by individuals, including habitual and novel foods. **a**, The cumulative mean number of unique food and beverage items consumed by each decile (~2,100 users per decile) over 14 days. **b**, The effects of sex on food diversity. **c**, Number of food and beverages consumed by any seven or more days by at least ~0.5% or >100 users. **d**, Mean number of items that constitute 50%, 75% or 90% of all records logged by a user. **e**, Mean number of times the highly preferred items are consumed by users in 14 days. Note, the 22nd item onward is almost always consumed only once (novel items). **f**,**g**, Frequency distribution of the timing of habitually consumed food and beverages (**f**) or novel items (**g**) shows that habitual items are preferred earlier, while novel items are more likely consumed at a later time in the 24 h day.

The subgroup-level differences in preferences for top-100 items suggests that individuals can differ in the diverse types of foods they eat and how frequently or regularly they eat these items. We considered the number of unique f/b consumed at least once during the 14-day period as the food diversity of each individual (20,982 participants after excluding participants who frequently used generic terms such as ‘breakfast’, ‘lunch’, ‘dinner’ and ‘snack’ in food logs and excluding common cooking ingredients and additives, including oils, condiments and sauces). From day 1 of the food logs (roughly comparable to one 24HDR), the average food diversity was 9.7 unique f/b (s.d., 4.74). The cumulative food diversity increased with each additional day of logging, reaching a mean of 33.84 unique f/b (s.d., 13.47) at the end of 7 days and a mean of 50.32 unique f/b (s.d., 20.21) at the end of 14 days. The bottom decile consumed 20 unique items and the top decile consumed 86 unique items (Fig. 6a and Supplementary Table 14). Next, we tested whether age, gender, work type or eating window affected food diversity. Food diversity was comparable between the bottom decile for the 95th percentile eating window (mean items, 48.21 ± 20.34; eating window, 10 h 54 m) and the top decile (mean items, 48.31 ± 19.39; eating window, 16 h 0 m). Food diversity was not greatly affected by age (mean number of items for <40 years, 49.82 ± 21.32; 40–60 years, 50.73 ± 19.74; >60 years, 50.09 ± 18.45) or work schedule (range from 48.13 to 51.34 items), but was markedly different between males and females, with females consuming almost ten additional unique items more than males (mean number of items for females, 53.45 ± 20.12; males, 44.88 ± 19.18) (Fig. 6b Supplementary Table 15).

Although an individual may consume a wide diversity of foods or beverages, only a few may be consumed regularly. To assess this pattern, we asked which f/b were consumed by at least 100 people (~0.5% of the cohort) for 14 days. No food was consumed by at least 0.5% of the cohort for all 14 days. Only four beverages were consumed by at least 100 people for all 14 days. Coffee was logged by 1,534 users for all 14 days; milk, black coffee and tea are the other three beverages consumed by at least 100 users for 14 days. After relaxing the criteria of regular consumption, we found that 108 items (75 foods and 33 beverages) were consumed by at least 100 people for ≥7 days (Fig. 6c and Supplementary Table 16). For example, the two most consistent items, coffee and tea, were consumed by 10,282 and 2,516 users for ≥7 days, respectively. As we have seen, each item has a typical TF50, so we asked for a given item whether the TF50 for regular consumers (reported for ≥7 days) is different from that of occasional consumers. Surprisingly, we found that for all 108 items, the TF50 from regular consumers is earlier than the TF50 of occasional consumers (reported for ≤2 days). For example, the TF50 of orange juice for regular consumers was 09:25 h while it was 12:15 h for irregular consumers. Other examples included milk (10:40 h, 13:20 h), black tea (10:57 h, 12:57 h), sandwich (12:15 h, 13:50 h), kombucha (13:54 h, 15:34 h) and pasta (14:37 h, 17:57 h).

**Fig. 7:**
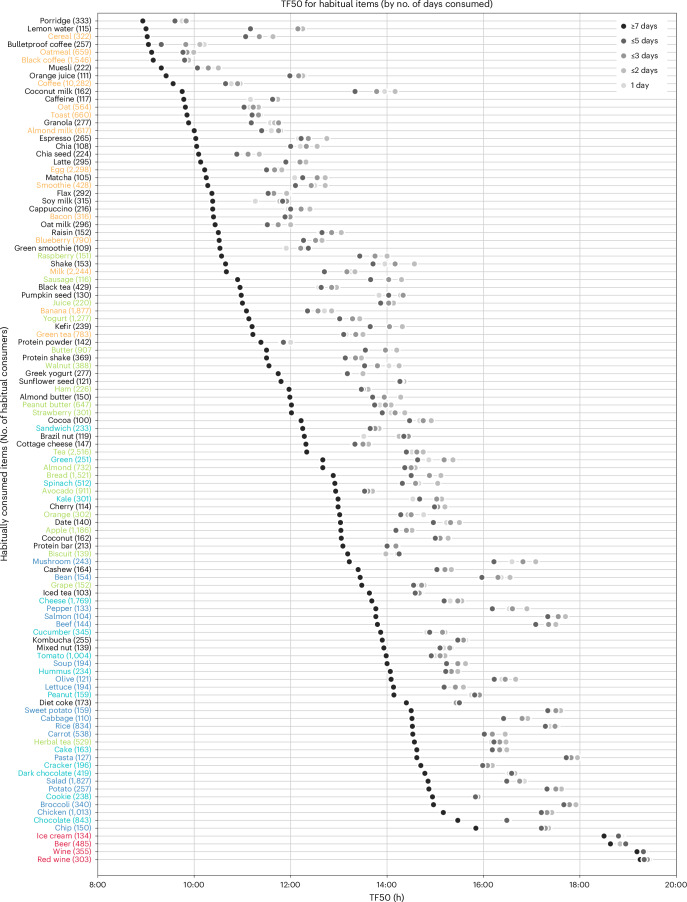
Early consumption of habitual items. TF50 of each of the 108 habitually consumed items. The name of the item and the number of participants who consumed it habitually (at least ten participants with ≥7 days of consumption out of 14 reporting days) and the median time of consumption in black circles are shown. Names of items are colour-coded if they were among the top-100 items in Fig. 4. The median time of consumption of the same items when consumed by at least 100 participants only once, ≤2, ≤3, ≤5 days out of 14 days are shown in grey. Notice that the black circles for most items are earlier than the grey circles.

As individuals can consume more than one item habitually, we found the average number of food and beverages consumed for ≥7 days was 3.85 items (s.d., 2.96). In other words, only approximately four out of ~51 unique items consumed in 14 days were consumed for ≥7 days. This approach does not account for whether the item is consumed exactly once per day for ≥7 days or more frequently on some days. On average, a user’s most frequently consumed item is logged 16.23 times, or the most preferred item is found in 16 logs. About half of a user’s unique items (mean, 25.66, s.d., 11.23) are found in more than one log, and the rest of the unique items were found only once in their logs. Dense ranking reveals that any items ranked past 22nd in frequency of consumption are consumed exactly once (s.d., 0) (Fig. 6e). This implies that a small subset of items typically accounts for a large share of an individual’s intake. We found that for 50% of users (out of the 21,000), only nine unique items are found in 50% of their logs. Similarly, 21 and 35 unique items are found in 75% and 90% of their logs, respectively (Fig. 6d and Supplementary Table 4). Frequency distribution of time of consumption of these novel foods (consumed only once) and habitual foods revealed that the novel consumption of foods was less likely to occur earlier in the day. As the day goes on, people are more likely to seek novel food and beverages (Figs. 6f,g and 7).

## Discussion

Temporal eating patterns have recently gained attention, with both perspective and intervention studies demonstrating the potential of such interventions in improving health. Diet diversity and timing are being linked to risks for several chronic diseases^14,59–65^, yet there is implicit acknowledgement that longitudinal dietary data can further refine such risk prediction^25^. The gold standard of nutrition research, 24HDR, continues to yield valuable epidemiological data on nutrition quality and quantity, yet there is both explicit and implicit acknowledgement of the need for multiday data collection, which faces significant logistics issues for scalability. With the increasing use of smartphones, smartphone-based nutrition monitoring and intervention are on the rise, with almost all of them focused on optimizing nutrition quality and quantity. We developed the mCC smartphone app for monitoring habitual or interventional daily eating patterns.

This study presents data from >20,000 adults who used the mCC app over a 10–14 day period, yielding one of the largest self-reported longitudinal datasets of eating behaviour to date. More than 93% of logs were recorded in the users’ smartphones in real time. The dataset enables fine-grained analysis of temporal eating behaviour and food preferences at both individual and population levels. At a population level, several metrics derived from this dataset align with those from large-scale surveys such as NHANES, including the average number of daily eating occasions and the timing of caloric intake. Notably, the dataset also allows the presentation of these metrics across percentile deciles, offering deeper insight into intra-population variability.

We present the temporal eating pattern with various metrics. The TF50 of an individual represents the midpoint of eating occasions but does not necessarily represent the median time of energy intake. Median time of energy intake has been used to describe early or late eating behaviours^52,53,66^, but does not report when the eating begins or ends. The average daily window, which averages the timespan between the first and last eating episodes, is typically reported in many chrononutrition studies^29,32^. However, day-to-day variation in first or last episodes can result in an artefactually short average window that does not account for ~25% of all eating occasions (Extended Data Fig. 1e). The 95th percentile eating window first described by our group^37^ by definition ignores only 5% of early and late eating occasions, but it also requires multiple days of dietary data and typically reports a eating window that is longer than what is reported by average window. By this measure, less than 24% of this cohort have an eating window of <12 h (Fig. 1f and Supplementary Table 6), while by average eating window, more than 80% of the cohort eats within ≤12 h (Extended Data Fig. 1c). Neither of these two windows report the day-to-day variations in the first and last eating occasions, which are better described by the average first-meal shift and average last-meal shift^54^. These two metrics revealed that only one in 20 participants maintains a consistent eating window during which the mean timing of first-calorie or last-calorie shift is <1 h over 2 weeks.

We also found an important relationship between the eating window and time of day. Irrespective of when the eating window begins, the eating window for >50% of users ended after 20:00 h (Fig. 1h), which is comparable with an earlier observation^67^. This tendency to consume food in the evening may reflect both circadian drive for evening hunger^68^ and the ancestral habit of socializing around food after the end of the day^69^. Accordingly, those who begin their eating window earlier in the day are likely to have a longer eating window, while those who start later have a shorter window that also ends after 20:00 h.

We also found important relations among food diversity, habitual foods and time of consumption. There is no universally accepted method to describe food diversity, consistency or regularity of food choices. One of the early methods of food diversity classified foods reported in a 24HR into five major food groups and assigned one point to each food group, resulting in a maximum score of five^13^. However, complex foods containing multiple components make this approach of oversimplifying foods into five groups challenging. Hence, other methods have classified foods into a larger number of groups and derived indices of healthy eating^15–17,70–74^. Heterogeneity of such classification systems has made it difficult to compare food diversity between studies. Our approach was to classify foods into usual food names that individuals can relate to. We find that food diversity continues to expand from fewer than ten on day 1 to 51 unique foods and beverages in 2 weeks. No single food item was habitually consumed every day over the 14 days. However, only a small number of foods were frequently logged; nine items were found in >50% of food records in 50% of users. Habitual foods were more likely to be consumed earlier in the day, and as the day progressed, the consumption of novel foods (items logged for <7 days) increased.

Observed temporal aspect of consumption of popular foods and of habitual versus novel foods can also interact with habitual eating windows, such that eating within a given time window may affect food preference. For example, a short eating window, as in TRE, that ends early or late in the evening can result in different food choices. Early TRE in which the eating window ends earlier is sometimes considered better, as it aligns the eating window with increased insulin sensitivity early in the day^75^. However, adopting an early TRE as opposed to a late TRE may also benefit from inadvertent improvement in diet quality. Adopting a late TRE can increase the likelihood of consumption of alcohol and energy-dense foods, such as ice cream, in the late evening. In support of this observation, a significant reduction in alcohol intake was noted in a TRE study on firefighters^41^.

We did not collect comprehensive health information or estimate energy intake, as is done in NHANES. Hence, we cannot make any definitive conclusions about temporal eating patterns and health. However, the dataset sheds important insights into how age, gender and work type affect food timing and food preferences. The working population of most countries, including the USA, comprises ~20% shift workers, who may have eating times or food preferences that differ from those of individuals who work regular hours. One study reported that ~20% of the NHANES cohort are shift workers^76^, and very few studies have compared the food habits of shift workers with those of individuals who work regular hours^76–78^. We found that shift workers were more likely to have a longer eating window and have more irregular first-meal and last-meal shifts. Yet the data also show that at least 20% of shift workers have a 95th percentile eating window of <12 h (Supplementary Table 6). We also found differences in eating patterns between sexes or between young and old participants, but these differences were smaller than those between shift and regular workers. For food diversity, the gender difference was larger than any other parameter; even participants who ate their food within a shorter or longer window had comparable food diversity. Such increased preference for diverse foods by females may reflect the known shopping habits of females preferring to purchase more diverse foods^79^. As more than 50% of the unique food items were consumed only once in 14 days, the food preferences of each subgroup are better revealed by the rank order of the top-100 frequently consumed items. In this metric, the rank order changed for almost 50% of items (47 out of 100) between young and old and relatively less by gender or shift work (37 out of 100 and 19 out of 100, respectively). In summary, the data also suggest that as one goes through life and chooses different types of work, what and when or how consistently one eats can also change. Furthermore, nearly 20% of adults at any given time are following a dietary intervention plan^80^, affecting what, how much or when to eat. All of these factors need consideration when making a correlation between dietary habits and current health status or predicting future health status based on current dietary habits. Conversely, the plasticity of dietary habits also offers an avenue to develop intervention plans to improve health.

This dataset is consistent with previous findings in much smaller datasets regarding the 95% eating window duration^37^ and meal frequency from NHANES data based on 2 days of dietary recall^81^. However, because of the large sample size (*n* > 20,000), real-time logging (93.54% of all logs) and longer eating assessment (10–14 days), it can uncover novel insights into temporal eating patterns.

### Limitations

The cohort comprises self-selected individuals who voluntarily used the app. They are more likely to be higher-educated, English speakers. The food logs did not have portion size information, and hence, the dataset lacks total energy intake information. Additionally, food diversity may be influenced by the participants’ food knowledge, their ability to disaggregate complex dishes (for example, salads) and social desirability bias in reporting. Given the name of the app, the users may also be aware of circadian rhythms in sleep–wake and feeding–fasting cycles and may have modified their eating pattern to have a shorter eating window than they had prior to use of the app. As with any self-reported timing of food intake, we cannot ensure that the time of intake reported is the actual time of consumption. In the absence of their habitual sleep time, sleep quality and physical activity pattern, it is difficult to fully interpret the physiological significance of eating time on health. Relatedly, the definition of daily activity taking place on a 04:00 h to 3:59:59 h basis was chosen to account for average human behaviour, yet some individuals—such as night-shift workers who have different sleep times—may be incorrectly characterized by this choice.

### Conclusion

This large-scale study that captured 10–14 days of participant dietary intake in real time found that both the timing and the nutritional components of dietary intake are highly variable. However, this method is also impractical for the collection of nutrition quantity at scale. Thus, newer approaches are needed that combine longitudinal food records with randomly selected in-depth reporting of portion sizes and detailed food descriptions to better assess dietary patterns from individuals to the population.

## Methods

### Study overview

This exploratory observational study was approved by the Salk Institute Institutional Review Board (15-0003). All dietary intake data were collected through the mCC app. The mCC app is a non-commercial research smartphone application designed primarily to collect the timing of dietary intake over several days or weeks^36^. It is available free of charge for all Android and iPhone smartphones. The app is designed to be used in multiple studies with study-specific and user-specific customizations. Specific custom versions of this app have been used in several lifestyle intervention studies^38–45,82,83^ in which participants meeting enrolment criteria used the app after in-person consent for the respective studies. For this study, the app was customized for web-based or in-app consent to collect dietary intake information from a large number of study participants.

During the app onboarding steps, user data (age, sex, education level, work type) were collected. Participants were asked to log their current lifestyle for 2 weeks. They received an automated daily reminder to log their dietary intake. During these 2 weeks, participants could not review their previous entries to minimize potential influence of reviewing records of dietary intake or timing on subsequent nutrition intake. At the end of the 2 week period, they could see all their entries and their calculated 95th percentile eating window. Hence, any data logged after the initial 2 weeks may have been influenced by the dietary feedback and were not used in the current analysis.

### Participants

Participants were recruited from 9 January 2015 to 19 February 2024. Adults (at least 18 years old) who could comprehend consent and adhere to recording daily nutrient consumption in English were included. There were no geographical restrictions on participation. Characteristics of the participants are provided in Supplementary Table 1. Participants were recruited using public tools such as flyers, social media posts and announcements of the study during public speaking opportunities. Participants registered to participate on the myCircadianClock website (www.mycircadianclock.org) or after downloading the mCC app. During the registration process, participants were provided with an electronic informed consent form. Upon consenting to participate, a user-specific access code for the mCC app was provided to activate the app on their smartphone. Participants were not compensated.

### Data collection

Data for this analysis were collected over a 2 week period to assess the current lifestyle of the users, with primary focus on eating patterns. Participants were asked to log all dietary intake, with options to also log water, medications, sleep, exercise and health metrics.

There were three types of food and beverage logs. Participants were asked to log their dietary intake at the beginning of the consumption event by (1) taking a picture of the item and providing a description of the food or beverage or (2) logging the description of the food or beverage without a picture. If they could not log at the time of consumption because they forgot or if they were in a social setting in which phone use may not be appropriate, they could (3) enter the description of the food or beverage and indicate the time and date of consumption. If the user’s phone did not have internet access at the time of recording, the time-stamped log waited in a queue until internet access could be obtained. All information was encrypted during transmission from the phone to the server. The time when the log reached the server was also recorded. The local time on the phone at the time of consumption (not the time of logging as in scenario 3 or the time of recording in the server) was used in all nutrition timing reported in this paper. Once logged, an item could not be deleted and the timing could not be changed.

### Data quality control

To be eligible for analysis, participants were required to meet logging compliance criteria for at least ten of their first 14 days using the mCC app. A logging day is considered compliant if it consists of two or more calorie-containing (water and non-caloric beverages excluded) eating occasions. A total of 26,115 participants met this criterion. Including an additional criterion of at least 5 h between the earliest and latest eating occasion excluded 5,109 additional users, resulting in 21,006 participants whose data were analysed further. Logging occasions were considered to belong to the same ‘compliance’ day for compliance calculation, beginning at 04:00 h on one calendar day and ending at 03:59:59 h the following calendar day. The log time used for compliance and analysis often differed from the server’s recorded log time. This occurred when the user forgot to log or could not log at the time of ingestion because of social circumstances. It also occurred when the user’s phone did not have internet access; in which case, logs were queued to be sent upon restoration of internet access. Any log with a time difference between the server-recorded reception time and the user-recorded ingestion time of greater than 15 min was considered to be a ‘delayed log’; 15.57% of logs were delayed logs. To distinguish between delayed logs caused by lack of internet access (which would still reflect accurate ingestion time) and ‘backlogs’ (logs for which the user relies on memory to approximate ingestion time), we examined the distribution of minutes for all delayed logs and found that minutes in multiples of five (0:00, 0:05, 0:15 and so on) exhibited preferential logging. A chi-squared test comparing the minute-level distribution of delayed versus real-time logs revealed a statistically significant difference between the two (*χ*^2^ = 59,298.74, df = 59, *P* < 0.001). Therefore, we considered any ‘delayed’ log occurring at minutes with large differences in logging frequency from expectation a ‘backlog’. Using this criterion, 41.49% of delayed logs were backlogs, accounting for a total of 6.46% of all data. To further verify data quality, we compared the measured statistics of the entire cohort against the remaining cohort after removing the 163 users with only delayed logs. We observed only marginal differences in the distribution of eating window metrics between the two cohorts.

Similarly, metrics that deal with food diversity or popularity exclude the following non-descriptive phrases that we observed to be frequently logged: ‘dinner’, ‘breakfast’, ‘lunch’, ‘snack’, ‘meat’, ‘protein’, ‘fruit’, ‘berry’, ‘seed’, ‘vegetable’, ‘appetizer’, ‘brunch’, ‘dessert’, ‘meal’, ‘leftover’ and ‘nut’. We examined the impact of disallowing non-descriptive food and beverage phrases for meeting compliance criteria and found that 876 users lost compliance without the allowance of non-descriptive phrases. We additionally examined excluding days during which the user was found to be compliant with a single unique item (for example, all logs for a day are the phrase ‘meals’). We compared excluding the 876 non-descriptive users and the additional 103 single-item users against the entire cohort and again found only marginal differences in the distribution of eating window metrics.

### Statistical methods

All statistical evaluations were conducted on distinct samples. All analyses, with the exception of permutation tests of JSD, were two-tailed. Kolmogorov–Smirnov and Levene’s tests were performed to examine the data for normality before selecting non-parametric tests. Following non-parametric analyses with multiple comparisons, post hoc pairwise between-group comparisons were performed using Dunn’s test as implemented in the scikit-posthocs Python package, which outputs a *P* value but does not yield a test statistic. All other statistical tests report both the test statistic and *P* value when appropriate. Effect sizes for non-parametric between-group comparisons were calculated using Cliff’s delta. The Wilcoxon signed rank test was used for within-group comparisons, with the rank-biserial correlation reported as the effect size. Chi-squared tests of independence were used to assess associations between decile membership for eating-window-related metrics and demographic qualities. Permutation testing was conducted for JSD using *n* = 1,000 permutations to determine whether the observed distance between subgroup item popularity distributions was significantly greater than expected by chance.

Exact *P* values are reported unless they are below the machine precision limit, in which case they are denoted as *P* < 0.0001.

### Item parsing

The food parser is an automatic text-processing pipeline designed to extract meaningful food, beverage and medication-related items from the mCC mobile app-based food logs. The software for log parsing was coded in Python with extensive use of NLTK for language processing. mCC app logs consist of one or more food, beverage or medication-related words or phrases written with the user’s natural language description. Individual food items or phrases within the same log are expected to be separated by a comma. Therefore, each log is split at its commas into multiple ‘sub-logs’, which are individually pre-processed by lowercasing and removing all special characters and punctuation. Commonly used English words (for example, ‘the’, ‘a’, ‘is’ and so on) and any numbers not immediately preceded or followed by a letter were also removed. Phrases that indicate amounts of measurements (for example, ‘oz’) are also removed. Any remaining words within each sub-log were converted to their root forms (for example, ‘eggs’ to ‘egg’). These words were compared against a dictionary of common food-related misspellings and abbreviations for spell correction. Instances in which known words were incorrectly joined were also identified and separated (for example, blueberrymuffin to blueberry muffin). The resulting processed sub-logs were then matched to our food item dictionary based on phrase length to find individual items within each sub-log (for example, single-word sub-logs were checked against single-word dictionary entries). Sub-logs were matched with food-related phrases of up to five words long, based on the total length of the sub-log. If no recognized phrases of that length were found, subsets of the sub-log were compared against dictionary items of increasingly shorter lengths.

### Food dictionaries

The food parser relies on the usage of two custom dictionaries: a food item dictionary and a typo dictionary. Users enter text descriptions of their food and beverage consumption. Common typos and abbreviations are changed into the assumed intention (for example, ‘coffeee’ to ‘coffee’) using a typo dictionary. Next, text descriptions are regularized into comma-separated lists of components using a food dictionary (for example, ‘Spaghetti,Salmon,Melted Cheese,Paprika’ to ‘spaghetti’, ‘salmon’, ‘cheese’ and ‘paprika’).

The typo dictionary consists of 2,336 common typos and abbreviations of items found in the food item dictionary.

The food item dictionary contains 5,396 phrases (consisting of one to five words), split into seven types: ‘foods’ (2,285), ‘beverages’ (542), ‘modifiers’ (964), ‘stopwords’ (547), ‘water’ (35), ‘medication’ (1,016) and ‘selfcare’ (7). Foods and beverages are individual descriptors of food and beverage items (for example, ‘muffin’ or ‘orange juice’). Modifiers and stopwords include adjectives and other modifying phrases (for example, ‘freshly squeezed’, ‘without sugar’) or unrelated words commonly found in food descriptions (for example, ‘nothing’, ‘eat’). Water includes water-related branding and phrases (for example, ‘dasani’, ‘tap water’). Medication includes medication and supplement-related terms (for example, ‘vitamin c’).

The typo and food dictionaries affect the outcomes of downstream analysis. The exact choices of regularizing food description here will directly affect measurements of food diversity and popularity.

### Logs and eating events

A participant log is any distinct entry made by an mCC app user that is recorded by our server. Logs are required to have a natural language description of the user’s intake for that specific log. No constraints on the style of description or the number of items within a log are made. Logs are assigned an original ‘log type’ (food, medication, beverage or water) by the user, indicating the majority of item types within a log. An ‘item’ is a singular, food parser-recognized word or phrase (for example, ‘hot dog’). Individual items are re-assigned an ‘item type’ based on our dictionary (for example, ‘avocado’ would be assigned ‘food’ regardless of the type of the log it is found in). A ‘caloric item’ is any recognized item that is designated as a ‘food’ or ‘beverage’. Water, club soda, seltzer and variants of sparkling water are considered ‘water’ and not a ‘beverage’. Non-caloric teas and black coffee are considered ‘beverages’ because of their impact on the fasting state. Any food or beverage items within logs made by the same participant without a 15 min or greater interval between log entry times are part of the same ‘eating event’. For example, caloric items within logs at 09:45 h, 09:56 h and 10:10 h belong to the same eating occasion, but additional items within a log at 10:26 h would be counted as part of a separate eating occasion. Water and other non-caloric items are not counted in eating events. Eating events are only calculated from logs within the same compliant day, beginning at 04:00 h on the first calendar day and ending at 03:59:59 h on the following calendar day. Definitions of various metrics used to describe temporal eating patterns or the regularity of foods are provided below.

### Time to 50% caloric log

The time of 50% of logging is calculated as the median log time for all food and beverage logs (excluding water) across the entire 14 day period.

### Time to 50% for food and beverages

The time of 50% for a specific food or beverage is calculated as the median log time for that specific food or beverage across the entire 14 day period.

### Average eating window

The average eating window is measured by calculating the average time between the earliest and latest caloric logs for each day on which the participant logged at least two caloric logs, with a minimum of 5 h between the first and last caloric log.

### 95% eating window

The mid-95th percentile eating window is calculated as the time window in which 95% of all caloric (food or beverage) items derived from all mCC logs were entered^37^. The earliest and latest 2.5% of caloric items are removed. Medication, water and other non-food or non-beverage descriptors are not included in calculating eating windows.

### Intake irregularity (first and last eating occasion shift)

The average shift in first and last caloric intake times measures the irregularity of a participant’s food habits. Intake shift was measured for the timing of both the first and last caloric events on each day during which the participant logged at least two caloric logs, with a minimum of 5 h between the first and last caloric log. Each measurement of shift is the absolute difference in time between a specific intake event occurring on successive days^54^. The first caloric intake shift is the shift in the timing of the first calorie consumed during the day, while the last caloric intake shift measures the shift of the last caloric intake event during the day.

### Food diversity and popularity

Food diversity is measured as the diversity of dictionary-matched caloric items logged by the participant. It excludes multiple common non-descriptive phrases: ‘dinner’, ‘breakfast’, ‘lunch’, ‘snack’, ‘meat’, ‘protein’, ‘fruit’, ‘berry’, ‘seed’, ‘vegetable’, ‘appetizer’, ‘brunch’, ‘dessert’, ‘meal’, ‘leftover’ and ‘nut’. Measurable food diversity may be impacted by a user’s preferred level of descriptiveness for their logged items and their ability or willingness to disassemble food items (for example, ‘salad’) into components and ingredients. As such, 141 ingredient-like items, including condiments, sauces and common cooking ingredients (for example, ‘salt’, ‘oyster sauce’), are not included in diversity calculations. Although user logging style is not controlled, consistency in verbosity is encouraged, and users can re-select the exact descriptors they have used previously when re-logging items. Food popularity is measured as the percentage of users who log an individual food or beverage item at least once.

### Habitual food

A ‘habitual food’ is a food or beverage consumed by at least 100 participants (~0.5% of the cohort) for at least seven out of the 14 days.

### Reporting summary

Further information on research design is available in the Nature Portfolio Reporting Summary linked to this article.

## Supplementary information

Supplementary Information Supplementary Tables 1–17 (excluding Supplementary Tables 4, 12 and 13).

Reporting Summary

Supplementary Data Supplementary Table 4: food/beverage logs and eating patterns of participants. Supplementary Table 12: rank order of food and beverages logged. Supplementary Table 13: effects of age, sex and work hours on the top-ranked food and beverages.

## Source data

Source Data Figs. 1–7 Each tab contains the respective data for the figures or figure subpanel.

Source Data Extended Data Figs. 1–3 Statistical source data.

## Data Availability

Deidentified participants’ eating patterns and timing of food consumption are presented in Supplementary Tables 4 and 12. Interactive visualizations of the data can be viewed at https://huggingface.co/spaces/tylrktran/eating_patterns. Source data are provided with this paper.

## Code availability

All code used to generate findings and figures is available at https://github.com/tktran11/eating_patterns. This repository includes a small sample set of data meant to illustrate the analysis workflow, but does not provide access to the full dataset.
