# Supplementary material for: The diversity and consistency of what and when people eat
Source: Nat Metab. 2026 Apr 23;8(4):981–97. doi: 10.1038/s42255-026-01504-0 (PMC13121034; doi:10.1038/s42255-026-01504-0)
Supplement: Supplementary file 1 — Supplementary Tables 1–17 (excluding Supplementary Tables 4, 12 and 13). [file 42255_2026_1504_MOESM1_ESM.pdf]

nature metabolism

Supplementary information

https://doi.org/10.1038/s42255-026-01504-0

The diversity and consistency of what and when people eat

In the format provided by the authors and unedited

Supplementary Materials

1. Tables 1-17
    i. Table S1: Participant characteristics
    ii. Table S2: Food/Beverage logs in hourly bins
    iii. Table S3: The daily average number of food/beverages logs and eating events logged
    iv. Table S4: Food/Beverage logs and eating patterns of participants…Separate File
    v. Table S5: Metrics to assess timing of daily eating patterns.
    vi. Table S6: Deciles of each eating pattern metric
    vii. Table S7: Effects of Age, Sex, Work Hours on Eating Pattern
    viii. Table S8: Statistical Tests for Work Hours
    ix. Table S9: Effect size for difference in eating pattern between users of self-reported work schedule.
    x. Table S10: 95% confidence interval for the difference in median eating pattern between users of self-reported work schedul
    xi. Table S11: Eating Pattern by Age
    xii. Table S12: Rank Order of Food and Beverages Logged …Separate File
    xiii. Table S13: Effects of Age, Sex, Work Hours on Top Ranked Food and Beverages.Separate File
    xiv. Table S14: Deciles of Food and Beverage Diversity
    xv. Table S15: Effects of Age, Sex, and Work Hours on Food Diversity
    xvi. Table S16. Consistency of food and beverage consumption: Number of food and beverages consumed by 100+ people for 7+ days
    xvii. Table S17. TF50 of Food/Beverages When Logged as a Habitual or Singleton Item

Table S1. Participant Characteristics.

	Male	Female	Unknown	Total
Participants who logged 10-14 days	7636	13327	43	21006
**Age**				
<40 yrs	3071	4732	27	7830
40-60 yrs	3724	7146	5	10875
>60 yrs	841	1449	0	2290
Unknown	0	0	11	11
**Work Hours**				
Regular Work Hours	3029	5099	6	8134
Flexible Schedule	2036	4387	17	6440
Long Work Hours	1169	1627	3	2799
Evening Shift	98	128	0	226
Morning Shift	182	272	1	455
Rotating shift	274	395	2	671
Night Shift	84	103	2	189
**Geographic Location**				
United States	4218	7519	33	11770
United Kingdom	476	1074	1	1551
Other	2107	2855	8	4970
Unknown	835	1879	1	2715

Table S2. Food/Beverage Logs in Hourly Bins. Count and percentage of food and beverage containing myCircadianClock logs in hourly bins.

Hour	Number of Logs	Percentage of All Logs
00:00	11142	0.42
01:00	5933	0.22
02:00	3662	0.14
03:00	3421	0.13
04:00	5630	0.21
05:00	17417	0.66
06:00	55278	2.08
07:00	120248	4.53
08:00	166861	6.28
09:00	175107	6.59
10:00	160551	6.05
11:00	170209	6.41
12:00	246752	9.29
13:00	212187	7.99
14:00	156551	5.89
15:00	131331	4.95
16:00	127533	4.8
17:00	176532	6.65
18:00	237167	8.93
19:00	202221	7.61
20:00	133511	5.03
21:00	77754	2.93
22:00	39592	1.49
23:00	19128	0.72

Table S3. The daily average number of food/beverages logs and eating events logged. Mean, SD, and 95% CI for daily log counts and eating events, grouped by deciles based on each participant's average.

	Individual Logs			Eating Events		
**Decile**	**Mean**	**SD**	**95% CI**	**Mean**	**SD**	**95% CI**
**1**	3.81	0.58	(3.78, 3.83)	2.76	0.27	(2.75, 2.77)
**2**	5.09	0.29	(5.08, 5.10)	3.32	0.12	(3.31, 3.32)
**3**	6.01	0.25	(6.00, 6.02)	3.68	0.1	(3.68, 3.68)
**4**	6.87	0.25	(6.85, 6.88)	4	0.1	(4.00, 4.01)
**5**	7.72	0.25	(7.71, 7.73)	4.33	0.1	(4.32, 4.33)
**6**	8.68	0.31	(8.67, 8.70)	4.66	0.1	(4.66, 4.67)
**7**	9.86	0.38	(9.85, 9.88)	5.03	0.11	(5.02 5.03)
**8**	11.38	0.51	(11.35, 11.40)	5.47	0.15	(5.46, 5.47)
**9**	13.64	0.84	(13.60, 13.67)	6.08	0.22	(6.07, 6.09)
**10**	19.18	3.75	(19.02, 19.34)	7.43	0.87	(7.39, 7.47)

Table S4: Food/Beverage logs and eating patterns of participants…………………Separate File

Table S5. Metrics to Assess Timing of Daily Eating Patterns. Definitions of metrics used to assess participant eating patterns.

	**Definition**	**Data Use**	**Units**
**TF50**	Median log time for all food and beverage (caloric) logs	All food and beverage containing logs per user	Clock Time (hh:mm)
**Avg. Eating Window**	Average time between earliest and latest caloric logs.	Days on which a participant logged 2+ caloric logs, >= 5h between first and last log.	Hours, minutes
**95% Eating Window**	Time window in which 95% of all caloric logs are logged.	All food and beverage containing logs per user	Hours, minutes
**Caloric Intake Shift**	Mean magnitude of day-to-day shift in the timing of a specific intake event, such as the first intake.	Days on which a participant logged 2+ caloric logs, >= 5h between first and last log.	Hours, minutes

Table S6. Deciles of Each Eating Pattern Metric. Chi-squared tests of independence were used to assess whether decile membership was dependent on demographic group. Table includes the percentage of each group per decile and the group-wise mean of the corresponding metric. P-values were corrected for multiple comparisons using the Holm-Bonferroni correction.

Decile	1	2	3	4	5	6	7	8	9	10	p value	χ^2	df
**95%ile window**	9h 50m	11h 23m	12h 07m	12h 40m	13h 10m	13h 38m	14h 07m	14h 42m	15h 27m	17h 50m			
% of all users	10	10	10	10	10	10	10	10	10	10			
% of all males in this decile	10.57	9.34	9.65	9.38	9.14	9.53	8.84	10.33	11.09	12.13	3.23056e-19	113.01	9
% of all Females	9.68	10.39	10.2	10.35	10.47	10.27	10.65	9.82	9.39	8.76			
% of all Regular workers	9.6	10.22	10.63	10.88	10.88	10.46	10.72	9.75	9.85	7.01	3.507097e-121	595.37	9
% of all Shift workers	7.46	6.94	6.88	6.55	7.46	7.01	9.99	9.73	11.16	26.8			
% of all young	9.96	9.8	9.72	9.68	9.9	10.1	10.23	9.64	9.57	11.4	7.996437e-07	48.57	9
% of all old	13.19	10.44	10.87	10.74	10.22	9.74	8.6	9.96	7.9	8.34			
**Beginning of 95% window (h)**	5.35	6.33	6.82	7.18	7.52	8.87	8.2	8.63	9.23	10.64			
% of all users	10	10	10	10	10	10	10	10	10	10			
% of all males in this decile	11.28	9.99	10.11	10.02	9.63	9.49	9.25	8.92	9.43	11.89	6.068905e-15	91.36	9
% of all Females	9.3	10.02	9.93	10.07	10.12	10.28	10.41	10.69	10.26	8.91			
% of all Regular workers	9.18	11.18	11.03	11.31	10.87	10.17	10.09	9.43	8.99	7.76	8.335985e-47	246.32	9
% of all Shift workers	21.61	11.1	8.76	7.79	7.79	7.66	7.33	9.02	8.18	10.77			
% of all young	7.78	8.3	8.51	8.94	9.4	10.1	10.64	11	12.12	13.22	3.831406e-32	175.99	9
% of all old	12.62	11.48	11.09	11.14	9.78	9.13	9.83	9.83	7.82	7.29			
**TF50**	11:12:00	12:25:00	12:54:00	13:16:00	13:39:00	14:05:00	14:33:00	15:10:00	16:02:00	17:54:00			
% of all users	10	10	10	10	10	10	10	10	10	10			
% of all males in this decile	11.52	9.89	9.25	9.53	9.26	8.8	9.56	9.68	10.36	12.15	4.781149e-21	122.18	9
% of all Females	9.12	10.07	10.41	10.29	10.42	10.67	10.27	10.19	9.78	8.76			
% of all Regular workers	11.06	12.34	11.41	10.73	10.4	9.39	9.22	8.84	8.75	7.84	2.032065e-15	227.9	9
% of all Shift workers	11.42	7.4	6.62	8.24	7.01	10.06	8.7	10.51	12.85	17.2			
% of all young	8.72	9.35	9.6	9.5	8.88	10.17	10.41	10.69	11.29	11.39	5.926095e-43	94.01	9
% of all old	12.88	10.13	10.09	10.7	12.31	8.43	8.43	7.99	9.13	9.91			
**Ending of 95% window**	18:20:00	19:27:00	20:01:00	20:28:00	20:53:00	21:18:00	21:45:00	22:17:00	23:03:00	01:07:00			
% of all users	10	10	10	10	10	10	10	10	10	10			
% of all males in this decile	9.72	9.42	9.73	9.48	9.26	9.65	9.63	10.01	11.08	12.04	8.668741e-14	85.26	9
% of all Females	10.19	10.32	10.29	10.2	10.43	10.17	10.4	9.84	9.35	8.81			
% of all Regular workers	10.61	11.46	11.34	11.02	11.05	10.5	10.44	8.96	8.45	6.18	5.779793e-151	734.1	9

% of all Shift workers	9.21	6.55	7.27	7.14	6.49	8.18	6.75	9.28	12.07	27.06			
% of all young	7.89	7.61	8.22	9.22	10.06	9.89	10.57	11.61	11.69	13.23	5.927338e-71	360.31	9
% of all old	16.99	13.67	11.18	9.91	9.39	7.82	8.3	7.29	8.25	7.21			
**First Caloric Intake Shift**	0h 33m	0h 49m	0h 59m	1h 09m	1h 18m	1h 28m	1h 40m	1h 54m	2h 14m	3h 07m			
% of all users	10	10	10	10	10	10	10	10	10	10			
% of all males in this decile	9.9	9.53	9.18	9.14	9.73	10.21	9.9	10.61	10.62	11.17	1.330057e-06	46.72	9
% of all Females	10.05	10.29	10.49	10.47	10.14	9.87	10.05	9.63	9.66	9.34			
% of all Regular workers	11.06	11.04	10.89	10.39	10.29	9.88	10	9.88	8.88	7.68	5.416378e-89	44.98	9
% of all Shift workers	5.52	6.75	7.07	8.83	7.92	8.05	8.89	10.12	13.24	23.62			
% of all young	10.57	9.94	9.11	9.89	10.17	9.92	9.91	9.69	10.06	10.74	0.06572738	16.06	9
% of all old	9.61	9.96	10.74	9.65	8.95	9.87	9.69	10.83	11.14	9.56			
**Last Caloric Intake Shift**	0h 45m	1h 05m	1h 18m	1h 29m	1h 41m	1h 53m	2h 05m	2h 21m	2h 43m	3h 36m			
% of all users	10	10	10	10	10	10	10	10	10	10			
% of all males in this decile	10.74	9.97	9.39	9.85	10.15	9.49	9.68	9.85	10.06	10.83	0.006811046	24.63	9
% of all Females	9.58	10.02	10.35	10.12	9.91	10.29	10.15	10.09	9.97	9.51			
% of all Regular workers	11.15	10.7	10.56	10.4	9.82	10.24	9.72	9.47	9.52	8.42	7.047941e-36	194.04	9
% of all Shift workers	6.29	6.16	7.27	8.5	9.6	10.25	10.45	11.68	12.72	17.07			
% of all young	8.94	9.11	9.71	10.08	10.2	10.1	10.63	10.28	10.61	10.34	2.233315e-07	52.03	9
% of all old	12.71	11.75	9.61	9.83	10.09	9.52	9.17	8.73	9.61	9			

Table S7. Effects of Age, Sex, and Work Hours on Eating Patterns. Mean (SD) of each eating pattern metric per self-reported demographic group.

	Participant Characteristics	TF50	95% Eating Window	Beginning of eating window	End of eating window	First food shift	Last food shift
Age	<40yrs	14:16 (116m)	13h 33m (137m)	8:01 (91m)	21:34 (115m)	1h 32m (48m)	1h 55m (49m)
	40-60yrs	14:02 (110m)	13h 30m (131m)	7:39 (86m)	21:08 (109m)	1h 30m (44m)	1h 53m (50m)
	>60yrs	13:57 (115m)	13h 16m (137m)	7:34 (85m)	20:48 (117m)	1h 31m (44m)	1h 49m (50m)
Sex	Male	14:14 (117m)	13h 36m (143m)	7:48 (95m)	21:24 (119m)	1h 34m (48m)	1h 54m (51m)
	Female	14:08 (100m)	13h 25m (129m)	7:46 (84m)	21:11 (110m)	1h 30m (44m)	1h 53m (49m)
Working Hours	Regular Work Hours	13:54 (107m)	13h 19m (118m)	7:41 (82m)	21:00 (100m)	1h 26m (41m)	1h 50m (47m)
	Flexible Schedule	14:14 (114m)	13h 17m (132m)	8:01 (89m)	21:17 (115m)	1h 30m (45m)	1h 53m (49m)
	Long Work Hours	14:11 (109m)	13h 52m (131m)	7:36 (89m)	21:28 (108m)	1h 35m (43m)	1h 57m (51m)
	Evening Shift	14:51 (130m)	13h 50m (144m)	8:23 (104m)	22:13 (125m)	1h 39m (54m)	2h 05m (44m)
	Morning Shift	13:30 (116m)	13h 53m (158m)	7:01 (96m)	20:54 (131m)	1h 41m (48m)	1h 58m (59m)
	Rotating Shift	14:38 (118m)	14h 45m (171m)	7:34 (96m)	22:19 (137m)	1h 55m (58m)	2h 12m (55m)
	Night Shift	16:39 (182m)	18h 04m (234m)	7:09 (140m)	1:13 (148m)	2h 58m (98m)	2h 49m (72m)

Average time or duration and (SD) are shown. TF50 = time to 50% of food/beverage logs.

Table S8. Statistical Tests for Work Hours. Kruskal-Wallis test assessed overall group differences in eating metrics. Post-hoc Dunn's tests were used for pairwise comparisons with Holm-Bonferroni correction.

Work Hours	TF50	95% Eating Window	First Eating Event Shift	Last Eating Event Shift
Kruskal Wallis Test Statistic	452.16	609.54	456.62	287.87
p-value	1.68E-94	2.04E-128	1.84E-95	3.25E-59

Post-hoc Dunn's Tests w/ Holm-Bonferroni Correction							
	Regular worker	Flexible schedule	Long work hours	Evening shift	Morning shift	Rotating shift	Night shift
**TF50 Statistical Tests**							
Regular Work Hours	N/A						
Flexible Schedule	3.82E-32	N/A					
Long Work Hours	4.99E-14	3.55E-01	N/A				
Evening Shift	4.80E-13	2.22E-05	5.76E-06	N/A			
Morning Shift	4.67E-04	3.67E-14	2.18E-11	3.00E-16	N/A		
Rotating Shift	1.15E-13	3.08E-06	4.99E-07	3.55E-01	6.48E-21	N/A	
Night Shift	6.39E-43	8.25E-28	1.09E-28	1.04E-06	5.31E-43	4.80E-13	N/A
**95% Eating Window Statistical Tests**							
Regular Work Hours	N/A						
Flexible Schedule	4.45E-02	N/A					
Long Work Hours	2.28E-31	1.66E-39	N/A				
Evening Shift	4.13E-02	6.75E-03	7.04E-01	N/A			
Morning Shift	4.44E-05	7.27E-07	8.24E-01	8.24E-01	N/A		
Rotating Shift	3.59E-37	1.73E-42	1.57E-08	5.97E-05	6.13E-06	N/A	
Night Shift	4.73E-58	8.22E-62	2.47E-34	7.38E-24	2.18E-28	2.98E-15	N/A
**First eating event shift statistical tests**							
Regular Work Hours	N/A						
Flexible Schedule	4.65E-05	N/A					
Long Work Hours	8.86E-19	3.67E-07	N/A				
Evening Shift	6.06E-03	1.25E-01	7.95E-01	N/A			
Morning Shift	1.53E-09	1.33E-05	1.25E-01	5.28E-01	N/A		
Rotating Shift	9.29E-37	1.91E-26	1.77E-12	6.84E-04	3.53E-03	N/A	
Night Shift	1.30E-52	1.16E-45	1.87E-34	1.61E-19	1.63E-20	6.74E-13	N/A
**Last eating event shiftstatistical tests**							
Regular Work Hours	N/A						
Flexible Schedule	1.55E-03	N/A					
Long Work Hours	9.07E-10	2.34E-03	N/A				
Evening Shift	2.84E-07	3.07E-05	4.49E-03	N/A			
Morning Shift	1.10E-01	1.00E+00	1.00E+00	4.49E-03	N/A		
Rotating Shift	6.44E-24	3.89E-17	2.92E-09	1.00E+00	3.24E-06	N/A	
Night Shift	4.10E-33	1.34E-28	1.30E-22	1.17E-06	7.49E-19	5.44E-08	N/A

Statistical tests for work hours were used to validate age and sex outcomes as values were similar.

Table S9. Effect size for difference in eating pattern between users of self-reported work schedule. Effect size of p-values reported in Table S8.

**Cliff's Delta for Effect Size Measurement**							
	**Regular worker**	**Flexible schedule**	**Long work hours**	**Evening shift**	**Morning shift**	**Rotating shift**	**Night shift**
**TF50**							
Regular Work Hours	N/A						
Flexible Schedule	1.17E-01	N/A					
Long Work Hours	1.01E-01	-1.74E-02	N/A				
Evening Shift	1.84E-01	1.84E-01	2.00E-01	N/A			
Morning Shift	-2.17E-01	-2.17E-01	-2.03E-01	-3.69E-01	N/A		
Rotating Shift	2.33E-01	1.2E-01	1.38E-01	-6.69E-02	3.22E-01	N/A	
Night Shift	5.66E-01	4.99E-01	5.12E-01	3.37E-01	6.20E-01	4.21E-01	N/A
**95% Eating Window**							
Regular Work Hours	N/A						
Flexible Schedule	-2.70E-02	N/A					
Long Work Hours	1.55E-01	1.73E-01	N/A				
Evening Shift	1.04E-01	1.25E-01	-4.27E-02	N/A			
Morning Shift	1.28E-01	1.46E-01	-1.49E-02	2.26E-02	N/A		
Rotating Shift	3.04E-01	3.15E-01	1.70E-01	1.99E-01	1.75E-01	N/A	
Night Shift	6.81E-01	6.80E-01	6.12E-01	6.15E-01	5.96E-01	4.91E-01	N/A
**First eating event shift**							
Regular Work Hours	N/A						
Flexible Schedule	4.36E-02	N/A					
Long Work Hours	1.17E-01	7.22E-02	N/A				
Evening Shift	1.25E-01	8.22E-02	1.39E-02	N/A			
Morning Shift	1.78E-01	1.35E-01	6.78E-02	4.98E-02	N/A		
Rotating Shift	2.97E-01	2.55E-01	1.93E-01	1.69E-01	1.25E-01	N/A	
Night Shift	6.39E-01	6.09E-01	5.76E-01	5.39E-01	4.23E-01	5.39E-01	N/A
**Last eating event shift**							
Regular Work Hours	N/A						
Flexible Schedule	3.61E-02	N/A					
Long Work Hours	8.27E-02	4.70E-02	N/A				
Evening Shift	2.19E-01	1.83E-01	1.31E-01	N/A			
Morning Shift	5.13E-01	2.54E-02	-1.97E-02	-1.48E-01	N/A		
Rotating Shift	2.41E-01	2.06E-01	1.56E-01	2.79E-02	1.71E-01	N/A	
Night Shift	5.96E-02	4.85E-01	4.43E-01	3.59E-01	4.45E-01	3.20E-01	N/A

Table S10: 95% confidence interval for the difference in median eating pattern between users of self-reported work schedule. 95% confidence interval of values reported in Table S8.

Difference of Medians in Hours (CI)							
	**Regular worker**	**Flexible schedule**	**Long work hours**	**Evening shift**	**Morning shift**	**Rotating shift**	**Night shift**
**TF50**							
Regular Work Hours	N/A						
Flexible Schedule	0.38 [0.34,0.46]	N/A					
Long Work Hours	0.35 [0.25,0.42]	-0.03 [-0.13, 0.02]	N/A				
Evening Shift	1.07 [0.71,1.43]	0.68 [0.31, 1.04]	0.72 [0.35, 1.08]	N/A			
Morning Shift	-0.30 [-0.45, -0.07]	-0.68 [-0.83, -0.47]	-0.65 [-0.80, -0.41]	-1.37 [-1.80, -0.95]	N/A		
Rotating Shift	0.74 [0.57, 0.92]	0.36 [0.18, 0.52]	0.39 [0.24, 0.60]	-0.33 [-0.71, 0.07]	1.04 [0.77, 1.27]	N/A	
Night Shift	3.12 [2.58, 3.60]	2.73 [2.20, 3.22]	2.77 [2.23, 3.25]	2.05 [1.40, 2.67]	3.42 [2.85, 3.95]	2.38 [1.88, 2.90]	N/A
**95% Eating Window**							
Regular Work Hours	N/A						
Flexible Schedule	-0.09 [-0.17, -0.01	N/A					
Long Work Hours	0.55 [0.45, 0.64]	0.64 [0.54,0.73]	N/A				
Evening Shift	0.28 [-0.07, 0.66]	0.37 [0.04, 0.75]	-0.27 [-0.62, 0.10]	N/A			
Morning Shift	0.56 [0.27, 0.76]	0.65 [0.35, 0.86]	0.01 [-0.26, 0.22]	0.28 [-0.16, 0.65]	N/A		
Rotating Shift	1.11 [0.88, 1.27]	1.20 [0.97, 1.36]	0.56 [0.32, 0.73]	0.83 [0.42, 1.23]	0.55 [0.25, 0.93	N/A	
Night Shift	5.38 [4.48, 6.14]	5.47 [4.58, 6.26]	4.83 [3.93, 5.55]	5.10 [4.18, 5.84]	4.83 [3.93, 5.55]	4.27 [3.30, 5.02]	N/A
**First eating event shift**							
Regular Work Hours	N/A						
Flexible Schedule	0.05 [0.03, 0.08]	N/A					
Long Work Hours	0.13 [0.10, 0.16]	0.08 [0.04, 0.10	N/A				
Evening Shift	0.12 [-0.01, 0.24]	0.07 [-0.07, 0.19]	-0.01 [-0.15, 0.11]	N/A			
Morning Shift	0.25 [0.14, 0.32]	0.20 [0.09, 0.27]	0.12 [0.02, 0.20]	0.13 [-0.02, 0.28]	N/A		
Rotating Shift	0.38 [0.27, 0.49]	0.32 [0.22, 0.42]	0.24 [0.13, 0.36]	0.25 [0.09, 0.41]	0.13 [-0.01, 0.27]	N/A	
Night Shift	1.37 [1.10, 1.75]	1.32 [1.04, 1.75]	1.24 [0.96, 1.59]	1.25 [0.93, 1.71]	1.12 [0.83, 1.57]	0.99 [0.69, 1.42]	N/A
**Last eating event shift**							

Regular Work Hours	N/A						
Flexible Schedule	0.04 [0.00, 0.08]	N/A					
Long Work Hours	0.11 [0.06, 0.16]	0.07 [0.02, 0.12]	N/A				
Evening Shift	0.30 [0.20, 0.46]	0.27 [0.16, 0.42]	0.20 [0.09, 0.36]	N/A			
Morning Shift	0.07 [-0.04, 0.20]	0.03 [-0.08, 0.15]	-0.04 [-0.15, 0.09]	-0.24 [-0.40, -0.07]	N/A		
Rotating Shift	0.29 [0.22, 0.38]	0.25 [0.18, 0.33]	0.18 [0.11, 0.28]	-0.01 [-0.17, 0.11]	0.22 [0.08, 0.35]	N/A	
Night Shift	0.89 [0.70, 1.00]	0.85 [0.66, 0.96]	0.78 [0.59, 0.92]	0.58 [0.32, 0.73]	0.82 [0.57, 0.97]	0.60 [0.38, 0.70]	N/A

Table S11. Eating Pattern by Age and Sex. Mean (SD) values for eating pattern metrics stratified by age group and sex.

	Sex	TF50	95% Eating Window	Beginning of eating window	End of eating window	First food shift	Last food shift
<40yrs	Male (n=3071)	14:23 (126m)	13h 35m (145m)	8:06 (99m)	21:40 (122m)	1h 35m (50m)	1h 55m (50m)
	Female (n=4732)	14:12 (108m)	13h 31m (132m)	7:59 (85m)	21:30 (111m)	1h 30m (46m)	1h 56m (48m)
40-60yrs	Male (n=3724)	14:06 (120m)	13h 40m (139m)	7:38 (91m)	21:17 (114m)	1h 33m (46m)	1h 55m (51m)
	Female (n=7146)	14:01 (104m)	13h 25m (127m)	7:39 (82m)	21:04 (107m)	1h 29m (43m)	1h 52m (49m)
>60yrs	Male (n=841)	13:50 (123m)	13h 26m (151m)	7:25 (88m)	20:51 (122m)	1h 33m (48m)	1h 48m (54m)
	Female (n=1449)	14:01 (110m)	13h 10m (128m)	7:39 (83m)	20:48 (113m)	1h 30m (42m)	1h 50m (47m)

Table S12. Rank Order of Food and Beverages Logged - Separate File. Logging frequency in 2-hour bins and statistics for foods and beverages ordered by user popularity

Table S13. Effects of Age, Sex, Work Hours on Top Ranked Food and Beverages – Separate File. Ranks of the 200 most-logged foods and beverages across age, sex, and work schedule groups.

Table S14. Deciles of Food and Beverage Diversity. Median daily diversity per days passed, grouped by deciles based on diversity at day 14

	Days													
**Deciles**	**1**	**2**	**3**	**4**	**5**	**6**	**7**	**8**	**9**	**10**	**11**	**12**	**13**	**14**
**1**	4	7	9	10	12	13	14	15	16	17	17	18	19	20
**2**	6	10	13	16	18	20	22	23	24	26	27	28	29	30
**3**	7	11	15	18	21	23	25	27	29	31	32	34	35	37
**4**	7	12	17	20	23	26	29	31	33	35	37	39	40	42
**5**	8	14	18	22	26	29	32	34	37	39	41	43	45	47
**6**	8	14	20	24	28	32	35	38	40	43	45	47	50	51
**7**	9	16	21	26	30	34	38	41	44	47	50	52	55	57
**8**	9	17	23	29	33	38	42	45	49	52	55	58	60	63
**9**	10	19	26	32	37	42	47	51	55	58	62	65	68	71
**10**	12	23	31	39	45	51	57	62	66	71	75	78	82	86

Table 15. Effects of Age, Sex, and Work Hours on Food Diversity. Mean (SD) food and beverage diversity and sample sizes across age, sex, and work schedule groups.

Demographics		Number of Participants	Average Unique Food Items	Standard Deviation
Age	<40yrs	7817	49.82	21.32
	40-60yrs	10868	50.73	18.45
	>60yrs	2289	50.09	20.42
Sex	Female	13321	53.45	20.12
	Male	7621	44.88	19.18
Work Hours	Regular Work Hours	8125	49.89	20.16
	Flexible Schedule	6434	51.05	20.39
	Long Work Hours	2796	50.49	20.00
	Evening Shift	226	48.13	16.96
	Morning Shift	455	48.91	19.97
	Rotating Shift	671	51.34	19.13
	Night Shift	189	50.28	20.03

Table S16.Consistency of food and beverage consumption: Number of food and beverages consumed by 100+ people for ≥ 7days. Food and beverage items consumed by at least 100 users on 7 or more days, with consistency summarized by days of consumption.

		Days							
Item Name	Food (f) or Beverage (b)	7+	8+	9+	10+	11+	12+	13+	14
coffee	b	10282	9567	8736	7733	6489	5075	3442	1534
tea	b	2516	2169	1862	1547	1260	943	651	327
egg	f	2298	1702	1282	911	586	380	186	76
milk	b	2244	1986	1740	1485	1218	934	619	311
banana	f	1877	1384	959	686	459	275	136	46
salad	f	1827	1178	758	472	261	133	63	17
cheese	f	1769	1234	835	557	369	218	111	39
black coffee	b	1546	1405	1254	1081	876	659	409	159
bread	f	1521	1157	874	642	453	265	144	44
yogurt	f	1277	933	677	457	310	182	92	30
apple	f	1186	793	545	361	227	136	67	17
chicken	f	1013	553	322	179	108	51	24	8
tomato	f	1004	692	459	290	162	88	45	14
avocado	f	911	639	431	262	166	98	42	14
butter	f	907	678	513	364	252	155	89	30
chocolate	f	843	566	371	242	144	78	35	15
rice	f	834	595	431	305	185	117	61	20
blueberry	f	790	581	428	308	192	122	62	20
green tea	b	783	621	481	389	264	179	111	42
almond	f	732	531	374	256	170	104	51	15
toast	f	660	454	317	214	143	91	42	11
oatmeal	f	659	500	388	288	211	138	90	32
peanut butter	f	647	466	328	208	146	85	51	13
almond milk	b	617	498	411	305	231	164	100	44
oat	f	564	451	347	262	193	120	60	19
carrot	f	538	352	220	141	82	48	17	1
herbal tea	b	529	422	328	242	172	114	64	32
spinach	f	512	368	249	152	88	49	24	8
beer	b	485	325	208	130	76	39	24	3
black tea	b	429	372	310	249	195	133	74	29
smoothie	b	428	327	250	172	109	72	34	10
dark chocolate	f	419	303	226	164	110	67	34	11
walnut	f	388	282	205	150	90	49	25	6
protein shake	b	369	281	211	155	107	70	36	16
wine	b	355	261	178	123	81	48	25	11
cucumber	f	345	222	148	94	51	30	11	3
broccoli	f	340	215	134	88	50	34	14	4
porridge	f	333	273	200	159	120	70	38	12
cereal	f	322	233	170	117	79	51	22	7
bacon	f	316	207	136	80	48	28	13	5
soy milk	b	315	269	228	177	142	117	77	40
red wine	b	303	207	149	89	43	24	9	0
orange	f	302	223	160	111	71	45	23	7
kale	f	301	207	146	102	62	30	18	4

Item Name	Food (f) or Beverage (b)	7+	8+	9+	10+	11+	12+	13+	14
strawberry	f	301	199	125	79	38	17	12	4
oat milk	b	296	250	207	166	130	99	72	38
latte	b	295	242	210	173	128	89	60	28
flax	f	292	241	195	148	103	71	42	17
granola	f	277	193	141	97	51	28	15	5
greek yogurt	f	277	206	144	101	68	38	19	6
espresso	b	265	231	202	173	138	95	63	23
potato	f	257	134	81	45	26	14	6	2
bulletproof coffee	b	257	227	195	164	131	96	56	19
kombucha	b	255	171	130	89	51	27	16	4
green	f	251	184	130	91	59	41	20	8
mushroom	f	243	152	107	76	49	27	12	6
kefir	b	239	198	155	117	80	44	21	8
cookie	f	238	141	90	56	30	19	7	3
hummus	f	234	153	99	58	36	20	12	0
sandwich	f	233	176	128	91	50	29	12	2
ham	f	226	138	93	65	41	20	9	4
chia seed	f	224	170	119	80	53	39	21	6
muesli	f	222	179	136	100	66	41	16	6
juice	b	220	164	134	97	71	41	25	7
cappuccino	b	216	182	151	118	86	62	32	10
protein bar	f	213	155	115	81	57	38	21	7
cracker	f	196	116	60	29	19	10	4	3
soup	f	194	117	80	49	33	18	8	4
lettuce	f	194	140	87	46	26	15	7	1
diet coke	b	173	138	114	81	64	43	29	18
cashew	f	164	109	77	46	32	19	7	2
cake	f	163	101	56	32	15	8	3	1
coconut	f	162	118	78	54	36	18	10	0
coconut milk	b	162	124	108	77	56	30	19	8
sweet potato	f	159	103	61	36	23	9	5	2
peanut	f	159	104	68	49	26	18	8	4
bean	f	154	93	59	42	29	14	5	1
shake	b	153	115	88	70	43	29	13	4
grape	f	152	100	65	32	19	9	4	0
raisin	f	152	108	77	49	29	19	11	4
raspberry	f	151	97	67	48	31	16	7	1
almond butter	f	150	108	76	50	31	21	7	1
chip	f	150	92	44	25	16	5	2	1
cottage cheese	f	147	95	67	44	31	20	6	1
beef	f	144	101	62	43	28	19	10	4
protein powder	b	142	118	94	75	53	35	22	7
date	f	140	99	68	47	23	14	6	1
mixed nut	f	139	98	69	44	27	17	8	2
biscuit	f	139	80	53	34	20	11	7	2

Item Name	Food (f) or Beverage (b)	7+	8+	9+	10+	11+	12+	13+	14
ice cream	f	134	69	37	23	10	3	0	0
pepper	f	133	86	58	39	25	17	11	5
pumpkin seed	f	130	97	67	44	26	18	7	3
pasta	f	127	62	29	14	8	5	2	1
sunflower seed	f	121	86	65	44	31	16	8	4
olive	f	121	72	42	20	13	10	6	2
brazil nut	f	119	87	58	45	29	19	8	2
caffeine	b	117	101	80	66	51	36	21	6
sausage	f	116	74	48	29	17	10	3	1
lemon water	b	115	85	75	59	41	29	20	7
cherry	f	114	79	55	32	23	15	12	5
orange juice	b	111	88	71	54	41	24	11	2
cabbage	f	110	68	43	23	11	4	2	1
green smoothie	b	109	89	71	53	33	21	14	2
chia	f	108	88	68	54	36	22	10	3
matcha	b	105	77	58	40	26	16	11	3
salmon	f	104	62	38	20	12	7	3	1
iced tea	b	103	81	56	44	33	21	11	6
cocoa	b	100	77	56	41	30	20	14	6

Table S17. TF50 of Food/Beverages When Logged as a Habitual or Singleton Item. Time of 50% logging for habitual items (consumed by 100+ participants on ≥7 days) compared to the same items when logged by at least 100 one-time (singleton) consumers.

Food/Beverage	Habitual	Singleton
porridge	8:56:00	9:58:00
lemon water	9:02:00	11:30:00
cereal	9:03:00	11:42:00
bulletproof coffee	9:05:00	
oatmeal	9:08:00	10:00:00
black coffee	9:11:00	9:58:00
muesli	9:20:00	10:40:00
orange juice	9:26:00	11:55:00
coffee	9:36:00	11:12:00
coconut milk	9:46:00	14:28:00
oat	9:50:00	11:21:00
toast	9:54:00	11:30:00
caffeine	9:54:00	11:52:00
granola	9:54:00	11:40:00
almond milk	10:02:00	12:04:00
espresso	10:06:00	13:21:00
chia seed	10:08:00	11:28:00
chia	10:08:00	12:47:00
latte	10:09:00	12:27:00
egg	10:15:00	11:49:00
matcha	10:16:00	13:13:00
smoothie	10:18:00	12:55:00
cappuccino	10:23:00	13:01:00
soy milk	10:25:00	12:19:00
bacon	10:27:00	12:00:00
flax	10:27:00	11:48:00
oat milk	10:28:00	12:00:00
raisin	10:33:00	13:17:00
raspberry	10:35:00	14:13:00
blueberry	10:35:00	12:31:00
green smoothie	10:38:00	12:32:00
milk	10:45:00	13:26:00
shake	10:48:00	15:00:00
sausage	11:00:00	15:00:00
pumpkin seed	11:00:00	14:40:00
black tea	11:02:00	13:04:00
juice	11:05:00	14:11:00
banana	11:09:00	13:00:00
yogurt	11:13:00	13:35:00
kefir	11:16:00	14:39:00
green tea	11:18:00	13:44:00
protein powder	11:30:00	11:34:00
protein shake	11:34:00	13:30:00
butter	11:34:00	14:30:00
walnut	11:39:00	14:18:00
greek yogurt	11:48:00	14:12:00
sunflower seed	11:59:00	14:30:00
almond butter	12:00:00	14:31:00
ham	12:01:00	13:36:00
avocado	12:58:00	13:42:00

F/B Continued	Habitual	Singleton
spinach	12:59:00	15:36:00
bread	13:00:00	15:35:00
kale	13:01:00	15:59:00
cherry	13:03:00	15:25:00
orange	13:05:00	14:20:00
apple	13:06:00	14:31:00
date	13:07:00	15:45:00
coconut	13:10:00	15:36:00
protein bar	13:14:00	14:26:00
mushroom	13:14:00	17:24:00
biscuit	13:18:00	13:40:00
cashew	13:27:00	15:33:00
bean	13:33:00	16:47:00
iced tea	13:41:00	14:32:00
grape	13:44:00	14:53:00
salmon	13:47:00	17:58:00
cheese	13:50:00	15:59:00
pepper	13:53:00	17:16:00
cucumber	13:58:00	15:31:00
kombucha	13:58:00	15:30:00
tomato	14:02:00	15:22:00
mixed nut	14:04:00	15:27:00
beef	14:09:00	17:38:00
soup	14:10:00	15:40:00
lettuce	14:11:00	15:32:00
hummus	14:13:00	15:42:00
peanut	14:20:00	16:08:00
olive	14:22:00	17:06:00
diet coke	14:28:00	15:24:00
sweet potato	14:33:00	17:55:00
cabbage	14:38:00	17:07:00
carrot	14:40:00	16:41:00
cake	14:40:00	16:32:00
rice	14:42:00	17:45:00
herbal tea	14:44:00	16:46:00
dark chocolate	14:55:00	16:52:00
cracker	14:57:00	16:22:00
salad	14:59:00	16:47:00
potato	15:01:00	17:40:00
broccoli	15:06:00	18:01:00
cookie	15:09:00	15:56:00
pasta	15:26:00	18:07:00
chicken	15:30:00	17:33:00
chocolate	15:34:00	16:37:00
chip	16:06:00	17:24:00
ice cream	18:35:00	18:50:00
beer	18:48:00	18:45:00
wine	19:14:00	19:25:00
red wine	19:19:00	19:18:00

97
