# Supplementary material for: The diversity and consistency of what and when people eat
Source: Nat Metab. 2026 Apr 23;8(4):981–97. doi: 10.1038/s42255-026-01504-0 (PMC13121034; doi:10.1038/s42255-026-01504-0)
Supplement: Supplementary file 2 — Reporting Summary [file 42255_2026_1504_MOESM2_ESM.pdf]

nature portfolio

Corresponding author(s): Satchidananda Panda

Last updated by author(s): Mar 6, 2026

Reporting Summary

Nature Portfolio wishes to improve the reproducibility of the work that we publish. This form provides structure for consistency and transparency in reporting. For further information on Nature Portfolio policies, see our Editorial Policies and the Editorial Policy Checklist.

Statistics

For all statistical analyses, confirm that the following items are present in the figure legend, table legend, main text, or Methods section.

n/a	Confirmed	
☐	☒	The exact sample size (*n*) for each experimental group/condition, given as a discrete number and unit of measurement
☐	☒	A statement on whether measurements were taken from distinct samples or whether the same sample was measured repeatedly
☐	☒	The statistical test(s) used AND whether they are one- or two-sided<br>*Only common tests should be described solely by name; describe more complex techniques in the Methods section.*
☒	☐	A description of all covariates tested
☐	☒	A description of any assumptions or corrections, such as tests of normality and adjustment for multiple comparisons
☐	☒	A full description of the statistical parameters including central tendency (e.g. means) or other basic estimates (e.g. regression coefficient) AND variation (e.g. standard deviation) or associated estimates of uncertainty (e.g. confidence intervals)
☐	☒	For null hypothesis testing, the test statistic (e.g. *F*, *t*, *r*) with confidence intervals, effect sizes, degrees of freedom and *P* value noted<br>*Give P values as exact values whenever suitable.*
☒	☐	For Bayesian analysis, information on the choice of priors and Markov chain Monte Carlo settings
☒	☐	For hierarchical and complex designs, identification of the appropriate level for tests and full reporting of outcomes
☒	☐	Estimates of effect sizes (e.g. Cohen's *d*, Pearson's *r*), indicating how they were calculated

Our web collection on statistics for biologists contains articles on many of the points above.

Software and code

Policy information about availability of computer code

Data collection	The myCircadianClock smartphone app was used to collect participant data. Electronic consent and demographic information was entered on the myCircadianClock website (www.mycircadianclock.org) or the myCircadianClock smartphone app. Data was collected on the mCC app versions 1.0 (Android and iPhone, 2015) through versions 16.9.94 (Android, 2024) and 7.5.3 (iPhone 2024).
Data analysis	Data was analyzed using custom code. Custom code is provided via https://github.com/tktran11/eating_patterns [will be made available prior to publication].

For manuscripts utilizing custom algorithms or software that are central to the research but not yet described in published literature, software must be made available to editors and reviewers. We strongly encourage code deposition in a community repository (e.g. GitHub). See the Nature Portfolio guidelines for submitting code & software for further information.

Data

Policy information about availability of data

All manuscripts must include a data availability statement. This statement should provide the following information, where applicable:

- Accession codes, unique identifiers, or web links for publicly available datasets
- A description of any restrictions on data availability
- For clinical datasets or third party data, please ensure that the statement adheres to our policy

Data will not be made available in public repository due to privacy issue. A sample dataset will be available along with software codes.

Research involving human participants, their data, or biological material

Policy information about studies with human participants or human data. See also policy information about sex, gender (identity/presentation), and sexual orientation and race, ethnicity and racism.

Reporting on sex and gender	We have used the term 'gender' throughout the manuscript. Participants self-reported their gender as male, female, or other when signing up to participate. The study was to include all willing adults who consented to use the app. Data has been provided for all participants and broken down by gender for reporting and analysis.
Reporting on race, ethnicity, or other socially relevant groupings	Participants self-reported race and ethnicity during enrollment by selecting one of the following options: Hispanic or Latino, White, Black/ African American, Native Hawaiian and other pacific islander, Asian, Others, Prefer not to answer.
Population characteristics	See above
Recruitment	Participants were recruited using public tools such as flyers, social media posts, and announcementsof the trial during public speaking opportunities.
Ethics oversight	This study was approved by the Salk Institute Institutional Review Board (15-0003).

Note that full information on the approval of the study protocol must also be provided in the manuscript.

Field-specific reporting

Please select the one below that is the best fit for your research. If you are not sure, read the appropriate sections before making your selection.

☐ Life sciences ☒ Behavioural & social sciences ☐ Ecological, evolutionary & environmental sciences

For a reference copy of the document with all sections, see nature.com/documents/nr-reporting-summary-flat.pdf

Behavioural & social sciences study design

All studies must disclose on these points even when the disclosure is negative.

Study description	This is a mixed-methods observational study that collected both qualitative and quantitative data.
Research sample	Participants were any adults (18 years and older) who had access to the myCircadianClock app (mCC, free to use) and were able to comprehend consent and adherence. The mCC app was available in English. There were no geographical restrictions. This was designed to be a representative sample of the population.
Sampling strategy	This study used convenience sampling by using public advertisements and accepting all participants that wanted to participate. Sample-size calculations were not performed prior to analysis. Data saturation was not considered. Sample size was deemed to be of adequate size compared to previous data sets in the field of nutrition.
Data collection	Data was collected using the smartphone app myCircadianClock. Participants logged data over a two week period on their cell phones in a free living setting. The research team was not present when participants entered the data.
Timing	Data was collected from June 30, 2015 to February 19, 2024.
Data exclusions	Participants were included in data analysis if they completed at least 10 of 14 days of food logging in the mCC app.
Non-participation	226,189 individuals consented to download the app and participate in the study, 127,456 participants either did not download the app or did not use the app, 77,727 participants logged at least 1 entry, but did not meet the logging criteria of 10 days of adherent logging (minimum of 2 items entered at least 5 hours apart), resulting in 21,006 participants whose data was used in data analysis. The other participants were removed to ensure data quality.
Randomization	Participants were not allocated into experimental groups.

Reporting for specific materials, systems and methods

We require information from authors about some types of materials, experimental systems and methods used in many studies. Here, indicate whether each material, system or method listed is relevant to your study. If you are not sure if a list item applies to your research, read the appropriate section before selecting a response.

Materials & experimental systems

n/a	Involved in the study
☒	☐ Antibodies
☒	☐ Eukaryotic cell lines
☒	☐ Palaeontology and archaeology
☒	☐ Animals and other organisms
☐	☒ Clinical data
☒	☐ Dual use research of concern
☒	☐ Plants

Methods

n/a	Involved in the study
☒	☐ ChIP-seq
☒	☐ Flow cytometry
☒	☐ MRI-based neuroimaging

Clinical data

Policy information about clinical studies

All manuscripts should comply with the ICMJE guidelines for publication of clinical research and a completed CONSORT checklist must be included with all submissions.

Clinical trial registration	This is not an interventional trial and does not have a Clinicaltrials.gov registration number.
Study protocol	The study protocol will be submitted as supplementary material.
Data collection	Data was collected from June 30, 2015 til February 15, 2024 via the myCircadianClock smartphone app.
Outcomes	This was an exploratory study that did not have predefined outcomes.

Plants

Seed stocks	N/A
Novel plant genotypes	N/A
Authentication	N/A
